# The involvement of RNA N6‐methyladenosine and histone methylation modification in decidualization and endometriosis‐associated infertility

**DOI:** 10.1002/ctm2.1564

**Published:** 2024-02-12

**Authors:** Xiang Lin, Yongdong Dai, Weijia Gu, Yi Zhang, Feng Zhuo, Fanxuan Zhao, Xiaoying Jin, Chao Li, Dong Huang, Xiaomei Tong, Songying Zhang

**Affiliations:** ^1^ Assisted Reproduction Unit Department of Obstetrics and Gynecology Sir Run Shaw Hospital Zhejiang University School of Medicine Hangzhou China; ^2^ Key Laboratory of Reproductive Dysfunction Management of Zhejiang Province Hangzhou China

**Keywords:** decidualization, endometrial stromal cells, endometriosis, EZH2, hypoxia, RNA N6‐methyladenosine

## Abstract

Defective decidualization of endometrial stromal cells (ESCs) in endometriosis (EM) patients leads to inadequate endometrial receptivity and EM‐associated infertility. Hypoxia is an inevitable pathological process of EM and participates in deficient decidualization of the eutopic secretory endometrium. Enhancer of zeste homology 2 (EZH2) is a methyltransferase which catalyses H3K27Me3, leading to decreased expression levels of target genes. Although EZH2 expression is low under normal decidualization, it is abundantly increased in the eutopic secretory endometrium of EM and is induced by hypoxia. Chromatin immunoprecipitation‐PCR results revealed that decidua marker IGFBP1 is a direct target of EZH2, partially explaining the increased levels of histone methylation modification in defected decidualization of EM. To mechanism controlling this, we examined the effects of hypoxia on EZH2 and decidualization. EZH2 mRNA showed decreased m^6^A modification and increased expression levels under hypoxia and decidualization combined treatment. Increased EZH2 expression was due to the increased expression of m^6^A demethylase ALKBH5 and decreased expression of the m^6^A reader protein YTHDF2. YTHDF2 directly bind to the m^6^A modification site of EZH2 to promote EZH2 mRNA degradation in ESCs. Moreover, selective *Ezh2* depletion in mouse ESCs increased endometrial receptivity and improved mouse fertility by up‐regulating decidua marker IGFBP1 expression. This is the first report showing that YTHDF2 can act as a m^6^A reader to promote decidualization by decreasing the stability of EZH2 mRNA and further increasing the expression of IGFBP1 in ESCs. Taken together, our findings highlight the critical role of EZH2/H3K27Me3 in decidualization and reveal a novel epigenetic mechanism by which hypoxia can suppress EM decidualization by decreasing the m^6^A modification of EZH2 mRNA.

## Graphical abstract

- Increased EZH2 and H3K27Me3 expression in endometrial stromal cells (ESCs) contributes to defective decidualization of endometriosis by targeting IGFBP1.
- Hypoxia impairs decidualization via up‐regulating EZH2 and H3K27Me3 expression.
- Increased expression of demethylase ALKBH5 enhances EZH2 mRNA stability by catalysing m6A demethylation under hypoxia.
- Reduced expression of reader protein YTHDF2 leads to up‐regulated EZH2 protein expression under hypoxia.
- EZH2 depletion in ESCs promotes decidualization in vitro and in vivo.

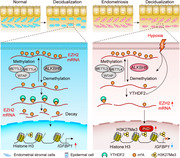

## 1 INTRODUCTION

Endometriosis (EM) is a common gynaecological disorder characterised by the presence of endometrial‐like gland or stroma outside the uterine cavity and causes dysmenorrhea, dyspareunia and/or infertility in 2–10% of reproductive aged women.^1^ EM occurs in 50% of women with infertility and is highly associated with unsuccessful outcomes of assisted reproductive technology because of decreased oocyte retrieval number, impaired endometrial receptivity and increased pregnancy complications.^2^ In‐depth study of the pathogenesis of EM‐associated infertility is crucial for improving infertility treatment outcomes for patients with EM and establishing new methods of targeted medical intervention.

Accumulating data suggest that the eutopic endometrium of women with EM shows defective decidualization and poor receptivity to embryo implantation.^3^, ^4^, ^5^ Decidualization involves the transformation of fibroblast‐like endometrial stromal cells (ESCs) into round and polynuclear decidual cells that provide a nutritive, immune‐privileged and temporal environment essential for embryo implantation.^6^ Decidualization of ESCs is characterised by genome‐wide chromatin reprogramming, markedly elevated expression of decidua markers (such as Insulin‐like growth factor‐binding protein‐1 (IGFBP1) and prolactin (PRL)) and terminal differentiation.^7^, ^8^, ^9^, ^10^, ^11^ After decidualization induction in vitro, ESCs present with a rounded epithelioid‐like phenotype and increased expression levels of IGFBP1 and PRL.^12^ IGFBP1 is the predominant protein expressed in decidual cells and is considered a specific biomarker of ESCs in decidualization.^13^, ^14^, ^15^ Hypoxia, a cellular stressor, plays a key role in EM,^16^ and hypoxia‑inducible factor‑1α (HIF‐1α) is expressed at higher levels in the eutopic endometrium of women with EM compared with that in the endometrium of those without during both the proliferative and secretory menstrual phases.^17^, ^18^ Moreover, hypoxia contributes to the deficient decidualization of EM, although the underlying mechanism remains unclear.^19^, ^20^

RNA N6‐methyladenosine (m^6^A) is the most common epigenetic nucleotide modification in eukaryotic mRNA molecules and can regulate mRNA stability, splicing, translation and export. M^6^A modifications are regulated by methyltransferases (writers), demethylases (erasers) and reader proteins.^21^ Numerous studies have shown the regulatory effects of hypoxia on epigenetic modifications.^22^ Emerging evidence suggests that m^6^A RNA methylation is involved in the post‐transcriptional stabilisation of hypoxia‐induced mRNAs.^22^ Furthermore, the promoter of the m^6^A demethylase ALKBH5 contains two putative binding sites for HIF‐1α.^23^ One study showed that m^6^A regulators METTL3, YTHDF2 and HNRNPA2B1 were significantly down‐regulated in the eutopic endometrium of EM patients compared with those in normal endometrium.^24^ However, no studies have examined if m^6^A RNA methylation modifications participate in decidualization of the eutopic endometrium from EM patients.

Histone methylation is another ubiquitous epigenetic modification that dynamically regulates active or inactive chromatin to support cell adaptation to hypoxic stress.^25^ Enhancer of zeste homology 2 (EZH2) is a methyltransferase that catalyses the trimethylation of histone 3 at lysine 27 (H3K27Me3), resulting in decreased expression of target genes.^26^ HIF‐1α transcriptionally activates EZH2 in various cells, leading to increased H3K27Me3 levels in target genes.^20^, ^27^, ^28^ EZH2 and H3K27Me3 expression levels were both elevated in endometriotic lesions from ovarian endometrioma and deep infiltrating EM compared with levels in control endometrium. This therefore may promote the development of EM via induction of the epithelial‐mesenchymal transition.^29^, ^30^, ^31^ However, the function of EZH2 in the eutopic endometrium of EM patients has not been determined.

In this study, we primarily focused on examining the role of hypoxia in m^6^A RNA methylation and histone methylation during decidualization. We identified EZH2 as a key downstream target of ALKBH5 under hypoxic conditions, with m^6^A modification of EZH2 mRNA increasing its stability in an YTHDF2‐dependent manner. Markedly increased EZH2 activity in ESCs reduced the expression levels of decidualization marker protein IGFBP1 through EZH2‐mediated catalysis of H3K27Me3 at the IGFBP1 promoter. Selective *Ezh2* depletion in mouse ESCs via *AMHRII‐Cre* can contribute to decidualization by increasing IGFBP1 protein expression levels, but does not affect expression of HIF‐1α, ALKBH5 or YTHDF2 during in vivo decidualization. In summary, we illustrate here that m^6^A RNA methylation and EZH2/H3K27Me3‐based histone methylation can work together in the deficient decidualization of EM and may contribute to EM‐associated infertility.

## 2 METHODS

### 2.1 Immunohistochemistry

Immunohistochemistry (IHC) was performed as previously described.^32^ Detailed IHC procedures are provided in the Supplemental Information and the primary antibodies used for IHC work are listed in Table 2.

### 2.2 Cell treatments, lentivirus infection and small interfering RNA

A variety of human ESC lines were included in this study. ESC‐1 through ESC‐13 lines were human primary ESCs extracted from the proliferative eutopic endometrium of 13 different EM patients. The hEM15A line is an immortalised ESC line from an EM patient.^33^ The THESC cell line is an immortalised normal human ESC line.

The isolation, purification, culture and authentication procedures for human primary ESCs were performed according to our previously described protocol.^34^ After culturing the cells for three passages, decidualization was induced by incubating the human ESCs in DMEM/F12 medium containing 2% FBS. Medroxyprogesterone acetate (1 μM) and 8‐bromo‐cAMP sodium salt (500 μM) (MPA + cAMP) co‐treatment for 72 h was used to induce decidualization of human ESCs in vitro.

Lentiviral vectors expressing a short hairpin RNA (shRNA) targeting EZH2 (Sh‐EZH2) or HIF‐1α (Sh‐HIF‐1α), lentiviral vector overexpressing EZH2 (LV‐EZH2) and the corresponding negative control lentiviruses (Sh‐NC or LV‐NC) were purchased from Genechem Co., Ltd. (Shanghai, China). A lentiviral vector overexpressing YTHDF2 (LV‐YTHDF2) and the corresponding negative control lentivirus (LV‐NC) were purchased from RiboBio (Guangzhou, China). ESCs were seeded in 6‐well plates at a density of 1 × 10^5^ per well and infected with lentivirus at a MOI of 10 for 48 h before decidualization induction. The medium was changed after 24 h of incubation, cells expressing fluorescence GFP were observed to evaluate infection efficiency. Western blots were used to confirm these observations (please see western blot section below).

ALKBH5 small interfering RNA (siRNA) (Si‐ALKBH5), YTHDF2 siRNA (Si‐YTHDF2) and the corresponding negative control siRNA (Si‐NC) were purchased from RiboBio. Following the manufacturer's instructions, 100 nM siRNA was transfected for 48 h to knock down target gene in human ESCs. Western blots were used to confirm knockdown efficiency at the protein level. The relevant shRNA, siRNA and LV sequences are listed in Table 3.

### 2.3 Chromatin immunoprecipitation and real‐time qPCR

We performed chromatin immunoprecipitation (ChIP) assays using the Simple ChIP® Enzymatic Chromatin IP Kit (#9003; Cell Signaling Technology (CST), Danvers, USA). Normal rabbit IgG (#2729; CST) and ChIP‐grade anti‐H3K27Me3 (#9733; CST) were used for IP (1 μg antibody per IP sample). ChIP‐enriched DNA were subjected to deep sequencing (ChIP‐Seq) and ChIP‐PCR was performed to verify the ChIP‐Seq results. ChIP‐Seq and bioinformatics analyses (volcano plot, KEGG and PPI analyses) were conducted at Biomarker Technologies (Beijing, China). ChIP‐PCR‐related primer sequences are listed in Table 1. 2% input was used in these experiments, and after optimising the ChIP‐PCR system, we use the comparative delta‐delta Ct method to analyse relative fold change.^35^ Fold change in occupancy was used to represent the differential occupancy fold change between the Sh‐NC and Sh‐EZH2 groups.

**TABLE 1:** List of primers used in qRT‐PCR, ChIP‐PCR, MeRIP‐qPCR and genome identification.

Gene name	Species	Sequence
*18S*	Human	Forward 5′‐CTCTTAGCTGAGTGTCCCGC‐3′
		Reverse 5′‐CTGATCGTCTTCGAACCTCC‐3′
*EZH2*	Human	Forward 5′‐ACATCCTTTTCATGCAACACC‐3′
		Reverse 5′‐TTGGTGGGGTCTTTATCCGC‐3′
*IGFBP1*	Human	Forward 5′‐GGCTCTCCATGTCACCAACA‐3′
		Reverse 5′‐CCATTCCAAGGGTAGACGCA‐3′
*PRL*	Human	Forward 5′‐AAGGATCGCCATGGAAAGCAG‐3′
		Reverse 5′‐GGTGGCAAGGGAAGAAGTGT‐3′
*MeRIP‐Primer 1*	Human	Forward 5′‐GCTGTTTCAGAGGAGGGGG‐3′
		Reverse 5′‐CCAGGCTGATGCCCTGAA‐3′
*MeRIP ‐Primer 2*	Human	Forward 5′‐TCAGGGTCACACTCTCGGAC‐3′
		Reverse 5′‐CAAAACCGCTTTCCGGGAT‐3′
*MeRIP ‐Primer 3*	Human	Forward 5′‐GCAGTTCTTGCAGGACACATTT‐3′
		Reverse 5′‐TCCGAGAGTGTGACCCTGAC‐3′
*MeRIP ‐Primer 4*	Human	Forward 5′‐CGCAATGAGCTCACAGAAGT‐3′
		Reverse 5′‐TGGAAAGAACGGAAATCTTAAACCA‐3′
ChIP‐PCR of H3K27Me3	Human	Forward 5′‐CTCCCAGCTGAGCACTTGTTA‐3′
		Reverse 5′‐GATTTCATACTGTTTGTCCCGTTGT‐3′
GP‐*AMHRII*	Mouse (Genome)	Forward 5′‐GCCTGCATTACCGGTCGATGC‐3′
		Reverse 5′‐CAGGGTGTTATAAGCAATCCC‐3′
GP‐*Ezh2*	Mouse (Genome)	Forward 5′‐CATGTGCAGCTTTCTGTTCA‐3′
		Reverse 5′‐CACAGCCTTTCTGCTCACTG‐3′

### 2.4 RNA isolation and quantitative reverse transcriptase PCR

We perform RNA isolation and quantitative reverse transcriptase (qRT)‐PCR using the RNA‐Quick Purification Kit 189 (RN001; Shanghai Yishan Biotechnology Co., Ltd., Shanghai, China). Detailed procedures are provided in the Supplemental Information, and relevant primer sequences are listed in Table 1.

### 2.5 Western blot

We use Image J software to analyse the protein band signal and intensity. Detailed western blot procedures described in the Supplemental Information. The primary antibodies are listed in Table 2.

**TABLE 2:** Antibodies used in immunofluorescence, western blot, immunohistochemistry and chromatin immunoprecipitation.

Antigen	Catalog number	Dilution in WB	Dilution in IHC	Dilution in IF/ChIP	Producer	Country
GAPDH	60004‐1‐Ig	1:10 000	Not applied	Not applied	Proteintech	USA
β‐Actin	66009‐1‐Ig	1:1000	Not applied	Not applied	Proteintech	USA
EZH2	5246	1:1000	1:50	1:100	CST	USA
H3K27Me3	9733	1:1000	1:200	1:200 for IF, 1:50 for ChIP	CST	USA
H3K9Me3	13969	1:1000	Not applied	Not applied	CST	USA
H3K4Me3	9751	1:1000	Not applied	Not applied	CST	USA
H3K36Me3	9763	1:1000	Not applied	Not applied	CST	USA
H3K79Me3	4260	1:1000	Not applied	Not applied	CST	USA
KDM6A	ab36938	1:1000	Not applied	Not applied	Abcam	UK
HIF‐1α	ab16066	1:800	1:100	Not applied	Abcam	UK
ALKBH5	ab195377	1:1000	1:150	Not applied	Abcam	UK
YTHDF2	80014	1:1000	1:100	Not applied	CST	USA
IGFBP1	sc‐55474	1:800	1:100	Not applied	SANTA	USA
FTO	31687	1:1000	Not applied	Not applied	CST	USA
METTL3	86132	1:1000	Not applied	Not applied	CST	USA
WTAP	56501	1:1000	Not applied	Not applied	CST	USA
PRL	ab188229	1:1000	Not applied	Not applied	Abcam	UK

**TABLE 3:** The sequence of Sh‐RNAs and Si‐RNAs.

Name	Targeting sequence
Sh‐EZH2	CAACATAGATGGACCAAAT
Sh‐HIF‐1α	TGTGAGTTCGCATCTTGAT
Sh‐NC	TTCTCCGAACGTGTCACGT
Si‐ALKBH5	ACAAGTACTTCTTCGGCGA
Si‐YTHDF2	GCGGGUCCAUUACUAGUAA
Si‐METTL3	GCUACCUGGACGUCAGUAU

The given sequence of negative control lentiviruses is the insertion sequences. The sequence information of all siRNA controls were from RiboBio (Guangzhou, China) and will not be disclosed.

### 2.6 Immunofluorescence

Immunofluorescence (IF) assays were performed in cells using a standard staining procedure, as described in the Supplemental Information. The primary antibodies used in IF are listed in Table 2.

### 2.7 Enzyme‐linked immunosorbent assay

Enzyme‐linked immunosorbent assays (ELISAs) were used to evaluate the human IGFBP1 (EK1255‐96; Multi Sciences Biotech, Hangzhou, China) and human PRL (EK1304‐96; Multi Sciences Biotech) concentrations in cell culture supernatants from the 13 human primary ESC lines described above. ELISAs were also used to evaluate the oestrogen and progesterone (25‐0175; ET Healthcare, Suzhou, China) concentrations in 14 paired serum samples from wild‐type (WT) or knockout (KO) mice at pregnant mare serum gonadotropin primed–48 h (PMSG‐48 h) and 15 paired serum samples from WT or KO mice at PMSG‐48 h followed by human chorionic gonadotropin (HCG) treatment for 4 h (HCG‐4 h) following the manufacturer's protocols.

### 2.8 Liquid chromatography‐tandem mass spectrometry

Six human primary ESC lines (ESC‐5 to ESC‐10) were cultured under normoxic conditions, hypoxic conditions, MPA + cAMP treatment or hypoxia and decidualization combined treatment for 72 h, then collected for liquid chromatography–tandem mass spectrometry (LC–MS/MS) analysis (Wuhan Matwell Biotechnology Co., Ltd., Wuhan, China).

### 2.9 Dual‐luciferase reporter assay

A putative m^6^A recognition site (GGACU) was identified in the EZH2 mRNA. WT or mutant sequences (in which the recognition site was mutated) were subcloned downstream of the luciferase gene in the Pmirglo dual‐luciferase vector. The original sequence of Pmirglo‐WT was AGCAGGGACTGAAAC and the corresponding altered sequence of Pmirglo‐Mut was AGCAGGGCCTGAAAC. Cells at a density of 1 × 10^5^ in six‐well plates were co‐transfected with WT or mutant EZH2 reporter vector and Si‐YTHDF2/Si‐NC. At 48 h post‐transfection, luciferase assays were performed using the Dual‐Luciferase Reporter Assay System (E1910) following the manufacturer's protocol.

### 2.10 Methylated RNA immunoprecipitation‐PCR analysis and actinomycin D treatment

Methylated RNA immunoprecipitation (MeRIP)‐sequencing was performed by Hangzhou Lianchuan Biotechnology Co., Ltd. (LC‐Bio, Hangzhou, China). MeRIP‐qPCR analysis was performed following the standard methods described in the product instructions. Briefly, total RNA samples isolated from human primary ESCs treated with normoxic or hypoxic conditions were fragmented into approximately 100 nt fragments using reagent (AM8740; Invitrogen, Carlsbad, CA, USA). Fragmented RNA was immune‐precipitated using an anti‐m^6^A antibody (MeRIP m^6^A Kit; Merck Millipore, Darmstadt, USA). Enriched m^6^A‐containing mRNA was sent to LC‐Bio for high‐throughput sequencing, and the results were validated by qPCR analysis. The sequences of primers are included in Table 1.

For actinomycin D assays, human primary ESCs were treated with 5 μg/mL actinomycin D (Merck) for 0, 2, 4, 6 or 8 h after basic treatment. EZH2 mRNA was analysed using qRT‐PCR.

### 2.11 Conditional KO mouse and artificially induced in vivo deciduoma

*AMHRII‐Cre* mice were purchased from the Model Animal Research Center (MARC, Nangjing, China) and *Ezh2* ^flox/flox^ mice were purchased from The Jackson Laboratory (#022616; Sacramento, CA, USA). Mice with ESC‐specific KO of *Ezh2* (KO mouse) were generated by crossing *AMHRII‐Cre* mice with *Ezh2* ^flox/flox^ mice. Briefly, *Ezh2* ^flox/flox^ female mice mated with *AMHRII‐Cre* male mice to generate *Ezh2* ^flox/+^; *AMHRII‐Cre* mice. *Ezh2* ^flox/+^; *AMHRII‐Cre* females and males have normal fertility. The *Ezh2* ^flox/+^; *AMHRII‐Cre* males were mated with *Ezh2* ^flox/flox^ females to generate *Ezh2* ^flox/flox^; *AMHRII‐Cre* mice. The *Ezh2* ^flox/flox^; *AMHRII‐Cre* females were defined as KO mice, and the *Ezh2* ^flox/+^; *AMHRII‐Cre* females were defined as WT mice. The concise breeding strategy is shown in Figure 6A.

Adult C57BL/6 female mice mated with fertile males to achieve pregnancy or with vasectomised C57BL/6 males to induce pseudopregnancy. We define the day of vaginal plug as day 1 (GD1); GD8 indicates 8 days after embryo implantation. Artificial decidualization was conducted in one horn by inject 80 μL of sesame oil into the lumen on day 4 of pseudopregnancy; the other un‐injected horn was left untreated as control.^36^ Then uteri were collected at 2, 4, 6 or 8 days after oil infusion. We the wet weights of the traumatised and control uterine horns immediately after the mice were sacrificed by cervical dislocation. Uterine tissue was collected from both horns for IHC analysis or cell extraction. For control and EM mice used for inducing in vivo decidualization, ovariectomy was performed 7 days after endometrium transplantation surgery.

### 2.12 Isolation, purification and culturing of mouse uterine ESCs

Uterine tissue contains many cell types (mainly stroma cells and epithelial cells), so we therefore needed to extract, separate and purify the ESCs from each mouse uterus. Mouse ESCs from day 2, 4, 6 or 8 of oil‐injected uterine horns were isolated by enzymatic digestion.^37^, ^38^ Briefly, uteri from oestrous mice were split, cut into 2 mm pieces and incubated with 0.1% trypsin (Cat. No. 0260; AMRESCO Inc., Solon, OH, USA) and 1.2 mg/mL dispase II (Cat. No. 04942078001; Roche Diagnostics, Basel, Switzerland) in HBSS (Sigma–Aldrich, St. Louis, Missouri, USA) for 1 h at 4°C, 1 h at 23°C and 10 min at 37°C. To remove the epithelial sheets from the cell suspension, we passing the cell suspension through a 100 μm nylon mesh. To isolate mouse ESCs, the samples were then incubated in HBSS containing 0.5% collagenase I (Invitrogen) and penicillin/streptomycin for 30 min at 37°C. We vigorously shaken the digested uteri and filtered it through a 40 μm nylon mesh. The resulting cell suspensions were washed with HBSS and centrifuged for 5 min at 500×*g*. The precipitated ESCs from three pseudopregnancy mice were frozen as one sample for western blot analysis.

### 2.13 Fertility assessment

Fertility tests were conducted as previously described.^32^ Briefly, 6‐week‐old WT (*n* = 15) or KO mice (*n* = 15) in oestrous were housed with 6‐week‐old fertile C57BL/6 male mice (2:1). The day of confirmed vaginal plugs was defined as day 1. The numbers of pups per female and pups per litter for each genotype were determined and described as mean ± standard error of the mean.

### 2.14 Establishment of an endometriotic mouse model

Forty‐two EM model mice were established as described previously.^32^ Of the 42 mice, one died from intestinal obstruction, 21 were used for mating with fertile males and 20 were used for inducing in vivo decidualization. Detailed information for establishing this EM mouse model is described in the Supplemental Information and Figure S4C described the concise procedure.

### 2.15 Statistical analysis

We use SPSS version 19.0 and Graph Pad Prism 5 software for statistical analysis. We use the Mann–Whitney *U*‐test or unpaired Student's *t*‐test for statistical comparisons between two groups. For the comparisons of continuous variables among groups, one‐way ANOVA followed by LSD tests were conducted. All experiments were repeated three times.

## 3 RESULTS

### 3.1 Secretory endometrium from EM patients shows increased EZH2 and H3K27Me3 expression, but decreased IGFBP1 and PRL expression

**FIGURE 1:**
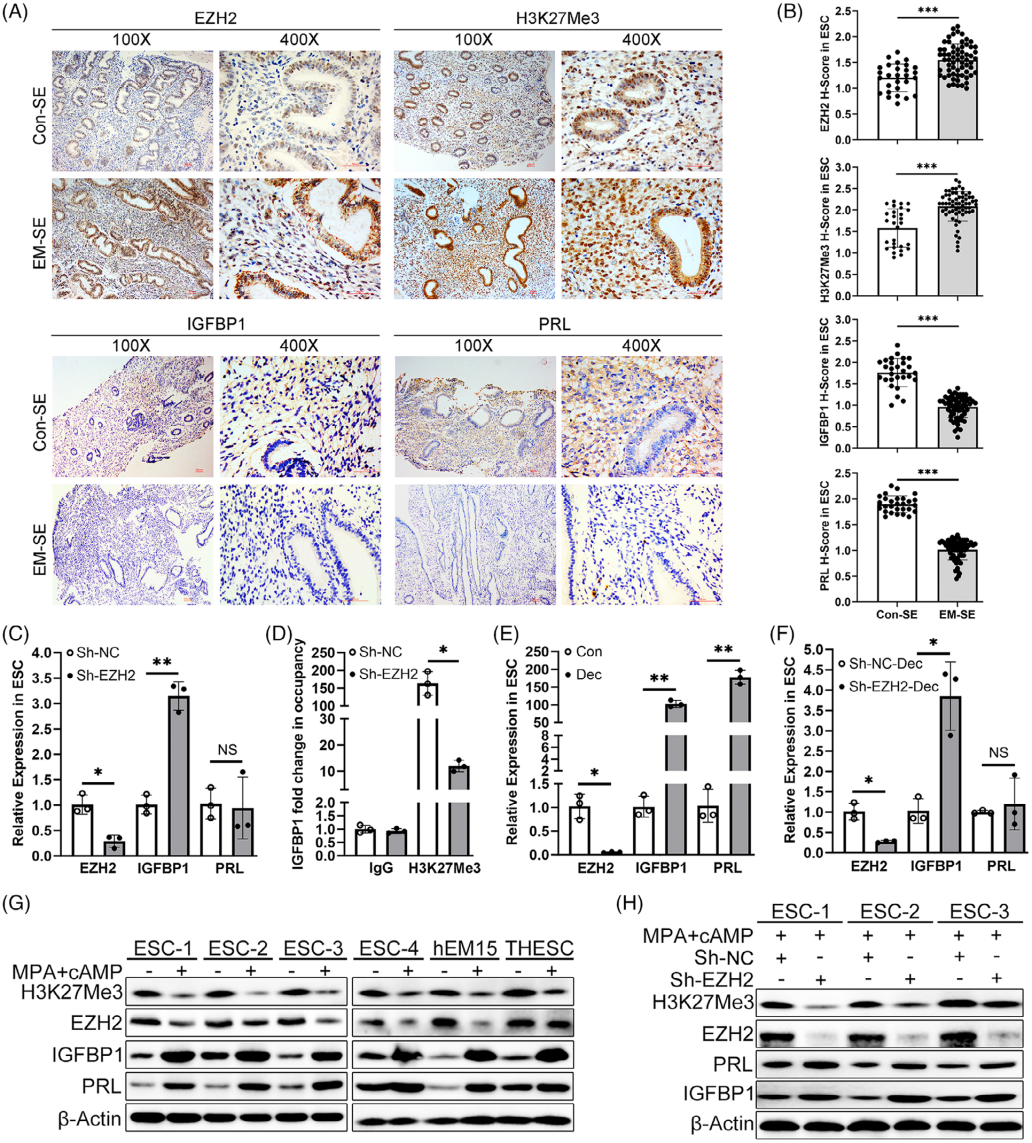
Histone methyltransferase EZH2 suppresses IGFBP1 expression by increasing H3K27Me3 modification on the IGFBP1 promoter. (A) IHC analysis of EZH2, H3K27Me3, IGFBP1 and PRL protein levels in human secretory endometrium (SE) from Con and EM patients (*n* = 28 for Con group and *n* = 64 for EM group). Scale bars = 50 μm, original magnification: ×100 or ×400. (B) The *H*‐score of EZH2, H3K27Me3, IGFBP1 and PRL in endometrial stromal cells (ESCs) of SE from Con and EM patients. Unpaired *t*‐test or Mann–Whitney test. (C) qRT‐PCR results of EZH2, IGFBP1 and PRL mRNA expression in human primary ESCs after transduction with lentiviral vectors expressing shRNA targeting EZH2 (Sh‐EZH2) or negative control (Sh‐NC) for 48 h. Paired *t*‐test. (D) ChIP‐PCR of IGFBP1 mRNA in human primary ESCs. Data are shown as mean ± standard deviation (SD) of three independent experiments. Paired *t*‐test. (E) qRT‐PCR results of EZH2, IGFBP1 and PRL mRNA expression in human primary ESCs after treatment with 1 μM medroxyprogesterone acetate and 500 μM 8‐bromo‐cAMP sodium salt (MPA + cAMP) treatment for 72 h to induce in vitro decidualization. Paired *t*‐test. (F) qRT‐PCR results of EZH2, IGFBP1 and PRL mRNA expression in human primary ESCs after treatment with Sh‐EZH2 or Sh‐NC for 48 h followed by decidualization induction for 72 h. (G) Western blot analysis of EZH2, H3K27Me3, IGFBP1 and PRL protein levels in human primary ESCs, hEM15A cells and THESC cells after MPA + cAMP treatment for 72 h. (H) Western blot analysis of EZH2, H3K27Me3, IGFBP1 and PRL protein levels in human primary ESCs after treatment with Sh‐EZH2 or Sh‐NC for 48 h followed by decidualization induction for 72 h. *p < .05, **p < .01, ***p < .001.

To explore the role of EZH2 and H3k27Me3 in the decidualization of EM, we analysed the expression levels of these proteins in secretory endometria (SE) from EM and control patients by IHC. Markedly higher EZH2 and H3K27Me3 protein expression levels were observed in the SE ESCs from EM patients compared with levels observed in SE ESCs from control patients (Figures 1A and B). The expression of EZH2 and H3K27Me3 were moderately in the nuclei of human ESCs, but positively expressed in both the nuclei and cytoplasm of human endometrial epithelial cells (EECs). In this study, we focused on expression changes in ESCs but not EECs. The *H*‐scores of ESCs are summarised in Figure 1B. IGFBP1 and PRL, the specific decidua markers of ESCs, were localised to the ESC cytoplasm. These markers were weakly expressed in the SE ESCs from EM patients compared with those in the SE ESCs from control patients (Figures 1A and B). No positive IGFBP1 or PRL IHC staining was found in the EECs of either the control or EM group (Figure 1A). The increased EZH2 and H3K27Me3 protein expression levels in SE and defective decidualization of the eutopic endometrium in EM patients implied a vital role of histone methylation modification in human ESC decidualization.

### 3.2 Histone methyltransferase EZH2 suppresses IGFBP1 expression by increasing H3K27Me3 modification at the IGFBP1 promoter

ChIP‐Seq was performed in human primary ESCs with EZH2 knockdown (Sh‐EZH2) or negative control treatment (Sh‐NC). The H3K27Me3 levels in the proximal regions near the IGFBP1 promoter (−305 to −170 bp from the transcriptional start site) were markedly less in the Sh‐EZH2 group compared with those in the Sh‐NC group (Figure S1A). qRT‐PCR results confirmed the reduced EZH2 and increased IGFBP1 mRNA expression levels in the Sh‐EZH2 group (Figure 1C). However, EZH2 knockdown did not influence the PRL mRNA expression levels (Figure 1C). ChIP‐Seq was conducted in three human primary ESCs with Sh‐EZH2 or Sh‐NC treatment, and the differentially expressed pathways enriched in the EZH2 knockdown group are summarised in Figure S1B. The enriched pathways, such as cytokine–cytokine receptor interaction, cell adhesion molecules, cAMP signalling pathway, neuroactive ligand–receptor interaction and calcium signalling pathway, are not only associated with EM pathogenesis,^39^, ^40^, ^41^, ^42^, ^43^, ^44^ but also may participate in the dysregulated decidualization of EM.^45^, ^46^ Further ChIP‐PCR results showed that there was reduced binding of the H3K27Me3 antibody to the IGFBP1 promoter in human primary ESCs with EZH2 depletion (Figure 1D).

MPA + cAMP co‐treatment for 72 h is the most commonly used method for inducing decidualization^7^ and was therefore used in our study. Normal decidualization is characterised by increased IGFBP1 and PRL protein expression and decreased EZH2 and H3K27Me3 protein expression (Figures 1E and G, ‘Dec’ is the abbreviation of decidualization induction via MPA + cAMP treatment). In EZH2 knockdown cells with decidualization induction (Sh‐EZH2‐Dec), we found markedly increased IGFBP1 mRNA and protein expression levels (Figures 1F and H).

PRL mRNA expression was not changed after EZH2 knockdown without decidualization induction (Figure 1C), and its expression levels remained unchanged in EZH2 knockdown cells with decidualization induction (Figure 1F). However, PRL protein expression was up‐regulated in EZH2 knockdown cells with decidualization induction (Figure 1H), though the mechanism remained unclear. These results suggest that EZH2 inhibition in human ESCs can lead to up‐regulated IGFBP1 expression through reduced H3K27Me3 modification at the IGFBP1 promoter. Modified histone methylation may be the underlying mechanism of defective decidualization in EM patients. However, the cause of aberrantly elevated EZH2 expression levels in ESCs from EM patients is unknown.

### 3.3 Decidualization is accompanied by decreased EZH2 expression, but hypoxia can increase EZH2 mRNA and protein levels

**FIGURE 2:**
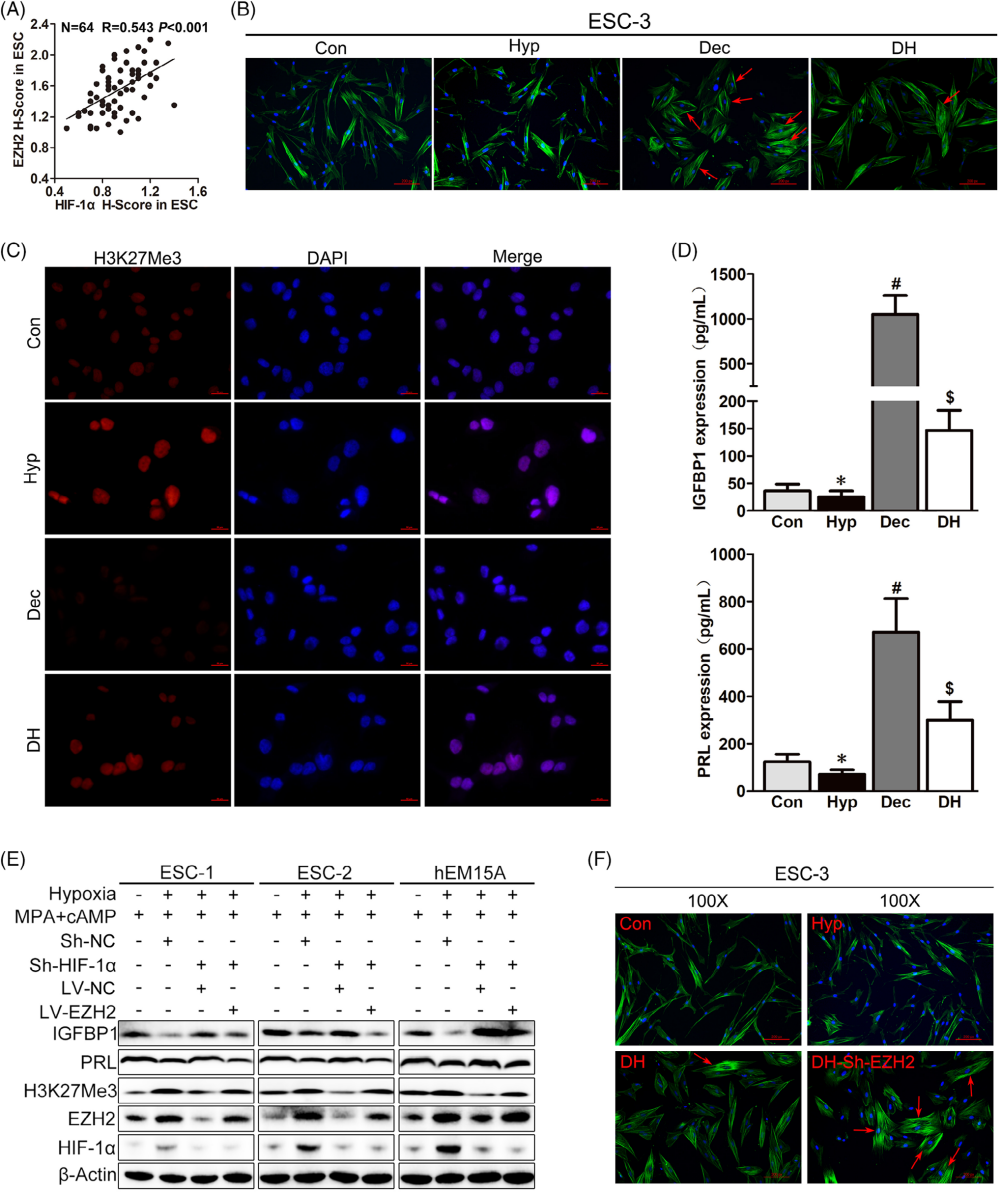
Hypoxia suppresses decidualization by up‐regulating EZH2 expression and reducing IGFBP1 expression. (A) Scatter diagram showing a significant *Pearson* correlation of EZH2 protein expression with HIF‐1α protein expression in human ESCs from the *H*‐score in ESCs from SE of EM patients (*n* = 64 for EM group). (B) Immunofluorescence (IF) analysis of fluorescence‐labelled phalloidine in human primary ESCs after normoxia culture (Con), hypoxic treatment (Hyp), decidualization induction (Dec) or hypoxia and MPA + cAMP combined treatment (DH) for 72 h. The red arrows refers to rounded epithelioid‐like decidual cells. Original magnification: ×200. (C) IF analysis in human primary ESCs shows the nuclear localisation and increased expression levels of H3K27Me3 after hypoxic treatment and reduced expression of H3K27Me3 after decidualization induction. DAPI (blue) was used to stain nuclei. Scale bar = 50 μm, original magnification: ×200. (D) ELISA results of IGFBP1 and PRL concentrations in human primary ESC supernatants (*n* = 11 for ESCs) after Con, Hyp, Dec or DH treatment for 72 h. Paired *t*‐test. (E) Western blot analysis of HIF‐1α, EZH2, H3K27Me3, IGFBP1 and PRL protein expression levels in human primary ESCs and hEM15A cells after treatment with MPA + cAMP for 72 h, hypoxia and MPA + cAMP combined treatment for 72 h, Sh‐HIF‐1α for 48 h followed by decidualization induction for 72 h, or Sh‐HIF‐1α for 48 h followed by LV‐EZH2 for 48 h and decidualization induction for 72 h. (F) IF results of phalloidine staining in human primary ESCs in the Con, Hyp, DH and DH‐Sh‐EZH2 groups. DH‐Sh‐EZH2, ESCs treated with Sh‐EZH2 for 48 h followed by decidualization induction for 72 h. The red arrows refers to rounded epithelioid‐like decidual cells. Original magnification: ×200.

HIF‐1α expression levels were increased in EM patient SE compared with those in control patient SE (Figure S1C), which was consistent with our previous studies.^33^, ^34^ Additionally, EZH2 *H*‐score showed a positive correlation with HIF‐1α *H*‐score in EM patient SE ESCs via linear regression analysis (*N* = 64, *R* = 0.543, *p* < .001; Figure 2A). Cell morphology changed from a long spindle type to round or polygon‐like shapes after decidualization induction, but no change was observed in cells cultured under hypoxic conditions (Figure 2B). IF results revealed that the morphological changes of primary ESCs from EM patients disappeared under hypoxia and MPA + cAMP combined treatment (DH) for 72 h, which suggests an adverse impact of hypoxia on decidualization (Figure 2B).

To further examine the exact influence of hypoxia on decidualization, we evaluated EZH2 and H3K27Me3 protein expression levels in human ESCs after normoxia culture (Con, 21% O_2_), hypoxic treatment (Hyp, 1% O_2_), decidualization induction (Dec) via MPA + cAMP treatment or hypoxia and MPA + cAMP combined treatment (DH). IF results showed EZH2 and H3K27Me3 protein were mainly localised to the ESC nucleus and accumulated there after hypoxia treatment for 24 h (Figures S1D and 2C). Moreover, EZH2 and H3K27Me3 protein expression levels were increased in the DH group when compared with levels in the Dec group (Figures S1D and 2C), although decidualization induction led to markedly decreased EZH2 and H3K27Me3 protein expression levels compared with levels in the Con group (Figure 1G). Remarkably elevated IGFBP1 and PRL concentrations in cell supernatants indicated the successful induction of decidualization (Figure 2D). ELISA results indicated that hypoxia could suppress the secretion of IGFBP1 and PRL by human primary ESCs. The IGFBP1 and PRL concentrations were also decreased under hypoxia and MPA + cAMP combined treatment (DH group) compared with those under MPA + cAMP treatment only (Figure 2D).

### 3.4 Hypoxia can impair decidualization by increasing EZH2 expression and decreasing IGFBP1 expression

ESCs were cultured under normoxic, hypoxic or MPA + cAMP treatment conditions for 72 h. Histone methylation modification can result in either positive or negative regulation of target gene expression through specific amino acid residues.^47^ To explore the spatiotemporal expression of activating histone modification marks (H3K4Me3, H3K36Me3 and H3K79Me3), repressive histone modification marks (H3K9Me3 and H3K27Me3), methylation transferases (EZH2) and demethylation transferases (KDM6A) in decidualization induction or hypoxic treatment, we examined their expression patterns via western blot. The levels of EZH2 and H3K27Me3 protein were decreased after decidualization induction, but increased after hypoxic culture (Figure S1E). No significant changes were observed for KDM6A, H3K4Me3, H3K9Me3, H3K36Me3 and H3K79Me3 levels (Figure S1E). Western blot results revealed that HIF‐1α, EZH2 and H3K27Me3 protein expression levels were increased in the DH group compared with levels in the Dec group (the second lane vs. the first lane, Figure 2E), which corroborated the IF results. Moreover, hypoxia and MPA + cAMP combined treatment reduced IGFBP1 and PRL protein expression compared with MPA + cAMP treatment only (Figure 2E), which was consistent with the ELISA results. Flow cytometry‐based cell cycle and apoptosis analyses were then conducted in human primary ESCs. Cell cycle distribution and cell apoptosis levels were appeared to be unchanged after decidualization induction compared with those in the control group (Figures S1F and G). However, the ratio of ESCs in the G2/M phase was up‐regulated in the DH group compared with that in the Dec group (Figure S1F), and cell apoptosis rates were markedly increased in the DH group compared with those in the Dec group (Figure S1G). After hypoxic culture for 72 h, the percentage of ESCs in S phase increased and human primary ESC apoptosis rates decreased (Figures S1F and G). These results demonstrate that decidualization induction under hypoxia can result in G2/M phase arrest and induce apoptosis, suggesting that hypoxia possibly interferes with normal decidualization and cell fate. The increased apoptosis rates in the DH group may be from the G2/M arrest of ESCs, but the involvement of EZH2 and the detailed mechanism require further research.

As summarised in Figure S2A, western blot analysis was used to confirm the infection efficiency of Sh‐HIF‐1α and Sh‐EZH2, as well as the knockdown efficiency of Si‐ALKBH5 and Si‐YTHDF2. ShRNA‐mediated knockdown of HIF‐1α (Sh‐HIF‐1α) during hypoxia and MPA + cAMP combined treatment decreased the expression of HIF‐1α, EZH2 and H3K27Me3 that was induced by hypoxia. It also rescued both IGFBP1 and PRL expression that were suppressed by hypoxia (the third lane vs. the second lane, Figure 2E). Furthermore, EZH2 overexpression (LV‐EZH2) after HIF‐1α silencing under hypoxia and MPA + cAMP combined treatment decreased IGFBP1 and PRL protein levels (the fourth lane vs. the third lane, Figure 2E). Consistent with the western blot results, EZH2 knockdown (Sh‐EZH2) under hypoxia and MPA + cAMP combined treatment restored the round or polygon‐like shapes of the decidual cells (Figure 2F). These results demonstrate that hypoxia can impair the normal decidualization of human ESCs by increasing EZH2 and H3K27Me3 expression levels and reducing decidua marker IGFBP1 expression, while EZH2 inhibition can effectively rescue hypoxia‐suppressed decidualization. These results indicate that suppression of decidualization by hypoxia is dependent upon EZH2 and H3K27Me3.

### 3.5 M^6^A demethylase ALKBH5 enhances EZH2 mRNA stability by catalysing m^6^A demethylation under hypoxia

**FIGURE 3:**
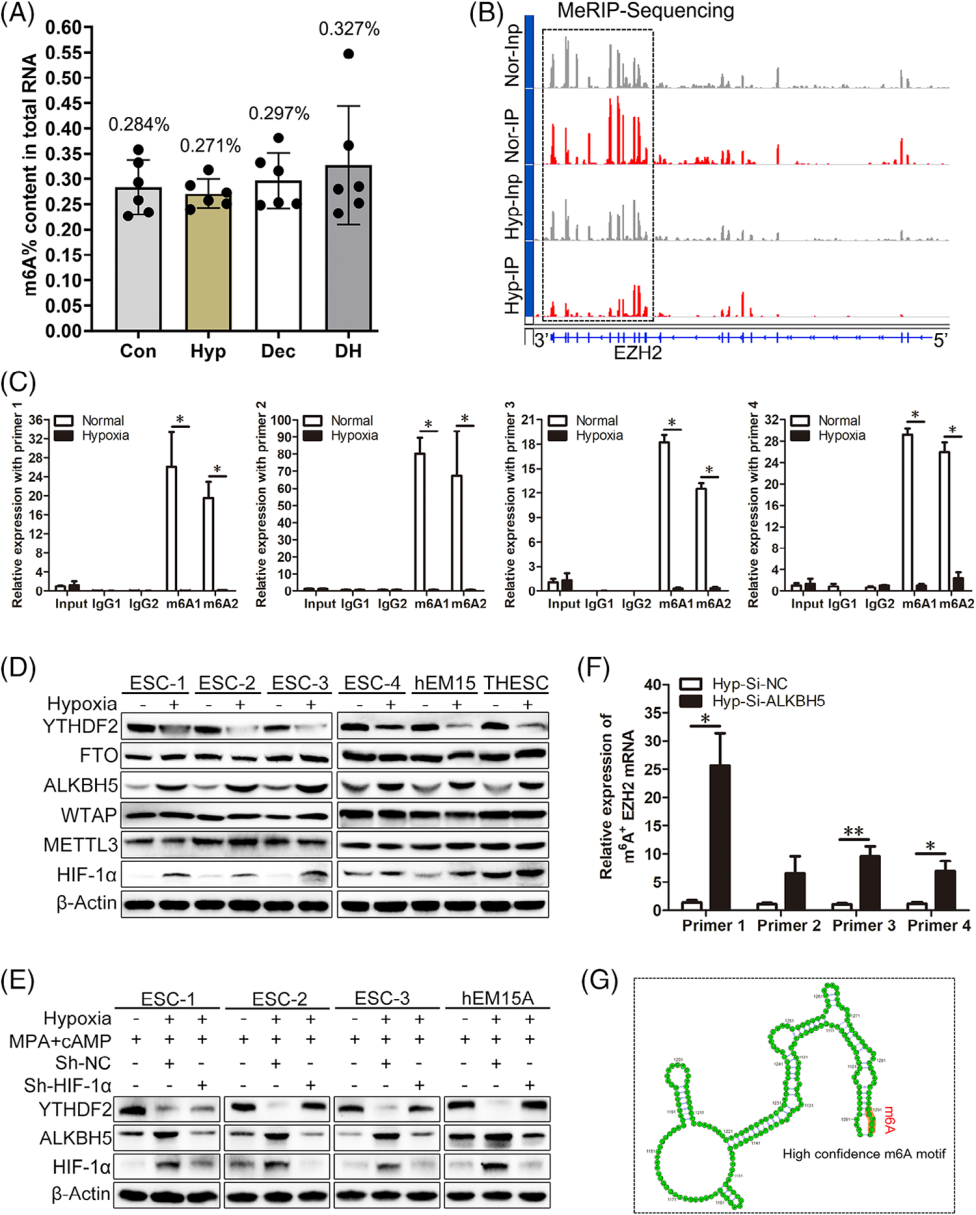
M6A demethylase ALKBH5 enhances EZH2 mRNA stability by catalysing m^6^A demethylation under hypoxia. (A) LC–MS/MS results showing the m^6^A percentage in total RNA from human primary ESCs after treatment with normoxia, hypoxia, decidualization induction or hypoxia and MPA + cAMP combined treatment for 72 h. Paired *t*‐test. (B) Integrative genomics viewer (IGV) was used to visualise MeRIP‐sequencing results of human primary ESCs treated with normoxia or hypoxia for 48 h. The input represents reads before immunoprecipitation (IP), and IP means enriched reads after IP using an m^6^A antibody. Nor, normoxia; Hyp: hypoxia. (C) MeRIP‐qPCR results showing the enrichment of EZH2 mRNA after IP with an IgG (IgG1 and IgG2) or m^6^A antibody (m^6^A1 and m^6^A2) using four different primers. Unpaired *t*‐test. (D) Western blot analysis showing the protein expression levels of HIF‐1α, METTL3, WTAP, ALKBH5, FTO and YTHDF2 in human primary ESCs, hEM15A cells or THESC cells after treatment in normoxic or hypoxic conditions for 48 h. (E) Western blot analysis showing the protein expression levels of HIF‐1α, ALKBH5 and YTHDF2 in human primary ESCs and hEM15A cells treated with MPA + cAMP for 72 h, hypoxia and MPA + cAMP combined treatment for 72 h, or Sh‐HIF‐1α for 48 h followed by hypoxia and MPA + cAMP combined treatment for 72 h. (F) MeRIP‐qPCR results showing the enrichment of EZH2 mRNA in human primary ESCs after transfection with a small interfering RNA targeting ALKBH5 (Si‐ALKBH5) or negative control (Si‐NC) under hypoxia for 48 h. Unpaired *t*‐test. (G) The site with the highest score that binds the 5′‐RRACU‐3′ m^6^A consensus sequence within the EZH2 mRNA via SRAMP sequence analysis.

During hypoxia, HIF‐1α binds to hypoxia response elements (HREs) of target genes and transcriptionally activates the expression of downstream genes.^48^ While HIF‐1α inhibition markedly reduced EZH2 and H3K27Me3 protein expression levels (Figure 2E), no HIF‐1α binding sites were found in the EZH2 promoter using multiple prediction methods. This suggests that the increased expression of EZH2 in ESCs under hypoxia was not regulated by the classical HIF‐1α/HRE pathway. Moreover, no HIF‐1α binding sites were found in the promoters of decidua marker genes using these prediction methods. Because hypoxic treatment or decidualization induction is accompanied by epigenetic modifications, we explored the overall m^6^A levels in total RNA by LC‐MS/MS. No significant differences in m^6^A levels were found between the Con, Hyp, Dec and DH groups (Figure 3A), but hypoxia could reduce the enrichment of m^6^A peaks on EZH2 mRNA through Integrative Genomics Viewer (IGV) analysis (Figure 3B). Figure S2B displays the peak density, which shows the metagene profile of m6A distribution. Further MeRIP‐qPCR work showed that use of an m^6^A‐specific antibody (m^6^A1 and m^6^A2) resulted in a marked enrichment of EZH2 mRNA compared with use of the IgG (IgG1 and IgG2) control antibody under normoxia (Figure 3C). However, the m^6^A enrichment level in EZH2 mRNA was reduced in the hypoxia group compared with the normoxia group (Figure 3C). These results suggest that hypoxic conditions decrease the m^6^A modification of EZH2 mRNA.

To elucidate the detailed mechanism of reduced m^6^A modification in EZH2 mRNA under hypoxia, we then examined the protein expression levels of m^6^A methyltransferases (writers), demethylases (erasers) and reader proteins. Western blot results showed that hypoxia could increase expression of eraser protein ALKBH5 and decrease expression of the reader protein YTHDF2, while METTL3, WTAP and FTO showed no expression changes in human ESCs (Figure 3D). Numerous articles have demonstrated that HIF‐1α can directly regulate ALKBH5 under hypoxia.^23^, ^49^, ^50^, ^51^ We also found four HIF‐1α binding sites in the ALKBH5 promoter using the JASPAR database (Figure S2C), suggesting that increased ALKBH5 expression in ESCs under hypoxia is potentially regulated by the classical HIF‐1α/HRE pathway. To explore the mechanistic link between hypoxia and ALKBH5, HIF‐1α was knocked down under hypoxic conditions. Our qRT‐PCR results showed that hypoxia could increase ALKBH5 mRNA expression levels, but had no influence on YTHDF2 mRNA expression (Figure S2D). ALKBH5 mRNA expression levels were decreased after HIF‐1α knockdown under hypoxia compared with those after hypoxia treatment only (Figure S2E). Western blot analysis also showed that HIF‐1α knockdown under hypoxia decreased ALKBH5 protein expression levels (Figure S2F). These results suggest a HIF‐1α‐dependent mechanism of ALKBH5 up‐regulation under hypoxic conditions. Moreover, the DH group showed increased ALKBH5 expression compared with the Dec group (the second lane vs. the first lane, Figure 3E), while HIF‐1α inhibition partly rescued the increased ALKBH5 protein expression induced by hypoxia (the third lane vs. the second lane, Figure 3E).

ALKBH5 inhibition under hypoxia reduced EZH2 mRNA expression levels (Figure S2G). Because ALKBH5 is a demethylase, we next examined if the altered EZH2 expression pattern is a result of ALKBH5‐mediated m^6^A methylation modification. IP experiments were performed on total RNA samples using an m^6^A antibody, then the immune‐precipitated RNA was used to amplify EZH2 mRNA using four different primers as described in Table 1. Silencing ALKBH5 under hypoxia could increase m^6^A methylation levels of EZH2 mRNA (Figure 3F), suggesting ALKBH5‐dependent m^6^A methylation. Sequencing analysis via a m^6^A modification site predictor (SRAMP) revealed 23 matches to the 5′‐RRACU‐3′ m^6^A consensus sequence within the EZH2 mRNA.^52^, ^53^ Four of these sites were considered very high confidence and eight were considered high confidence. The site with the highest score is shown in Figure 3G.

### 3.6 M^6^A reader protein YTHDF2 can suppress EZH2 protein expression by increasing EZH2 mRNA decay under hypoxia

**FIGURE 4:**
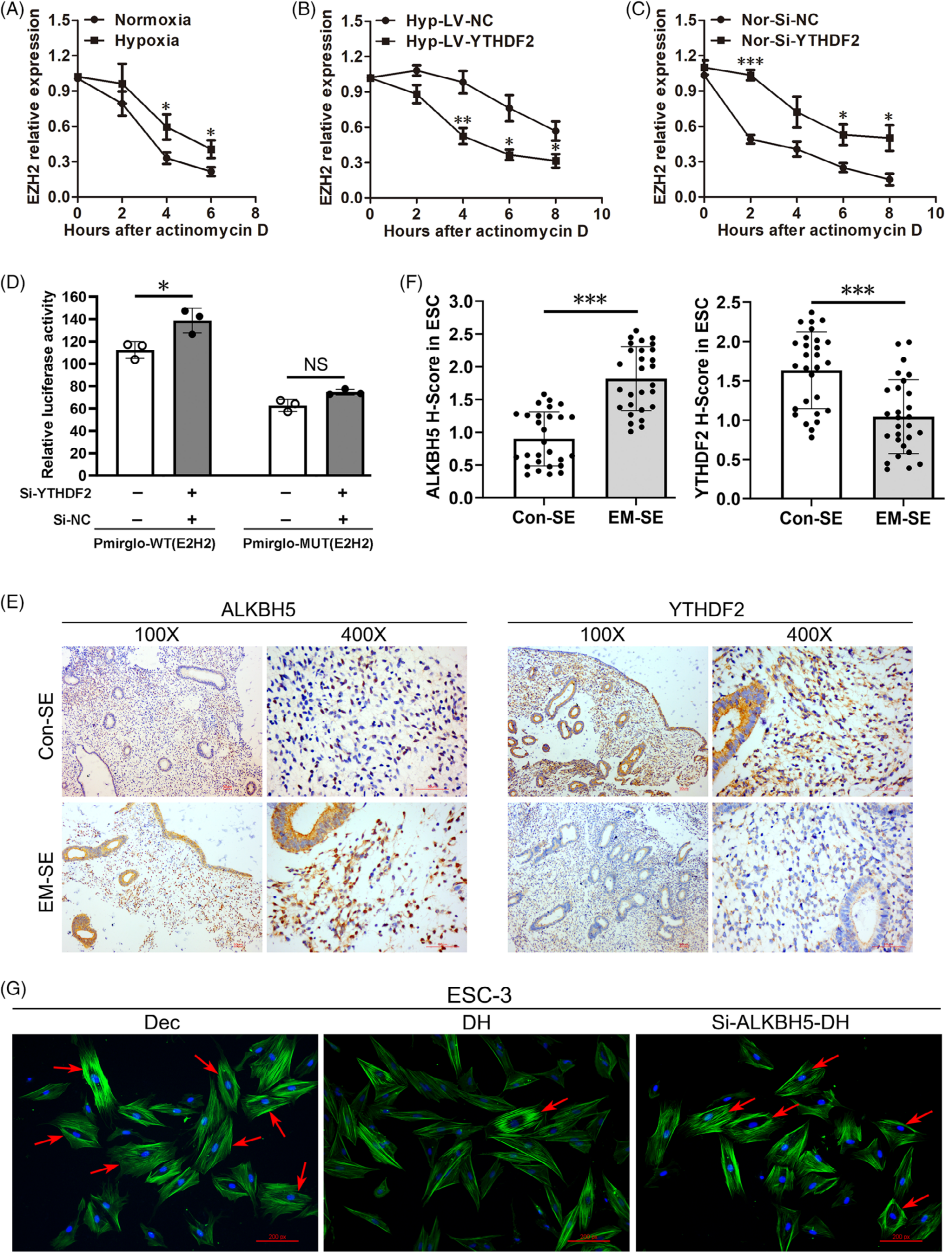
M6A reader protein YTHDF2 suppresses EZH2 protein expression by increasing EZH2 mRNA decay under hypoxia. (A) qRT‐PCR results of EZH2 mRNA expression levels in six human primary ESC lines after culturing under normoxic or hypoxic conditions for 48 h followed by 5 μg/mL actinomycin D treatment for 0, 2, 4, 6 or 8 h. Unpaired *t*‐test for each time point. (B) qRT‐PCR results of EZH2 mRNA expression levels in six human primary ESC lines after treatment with LV‐YTHDF2 or LV‐NC for 48 h under hypoxia followed by 5 μg/mL actinomycin D treatment for 0, 2, 4, 6 or 8 h. Unpaired *t*‐test for each time point. (C) qRT‐PCR results of EZH2 mRNA expression levels in six human primary ESC lines after treatment with Si‐YTHDF2 or Si‐NC for 48 h under normoxia followed by 5 μg/mL actinomycin D treatment for 0, 2, 4, 6 or 8 h. Unpaired *t*‐test for each time point. (D) Dual‐luciferase reporter assay results in THESC cells. THESC cells were transiently transfected with a luciferase reporter containing wild‐type (WT) or mutant (MUT) EZH2 3′ untranslated region (UTR), along with a short hairpin interfering RNA targeting YTHDF2 (Si‐YTHDF2) or negative control (Si‐NC). One‐way ANOVA. (E) IHC analysis of ALKBH5 and YTHDF2 in SE from Con and EM patients (*n* = 28 for Con group and *n* = 28 for EM group). Scale bars = 50 μm, original magnification: ×100 or ×400. (F) The *H*‐score of ALKBH5 and YTHDF2 in ESCs of SE from Con and EM patients. Unpaired *t*‐test. (G) IF analysis of phalloidine staining of human primary ESCs after decidualization induction (Dec) for 72 h, hypoxia and MPA + cAMP combined treatment (DH) for 72 h, or Si‐ALKBH5 for 48 h followed by hypoxia and MPA + cAMP combined treatment (Si‐ALKBH5‐DH) for 72 h. The red arrows refers to rounded epithelioid‐like decidual cells. Original magnification: ×200.

Although hypoxia can significantly decrease YTHDF2 protein expression (Figure 3D) and HIF‐1α inhibition under hypoxia can significantly up‐regulate YTHDF2 protein expression (Figure S2F), these conditions did not affect YTHDF2 mRNA expression levels (Figures S2D and E). To explore if YTHDF2 is involved in EZH2 mRNA decay, actinomycin D was added to the cell culture medium. Hypoxia could increase the half‐life of EZH2 mRNA in human primary ESCs compared with normoxia (Figure 4A). Although hypoxia dramatically delayed EZH2 mRNA degradation compared with normoxia, YTHDF2 overexpression under hypoxia (Hyp‐LV‐YTHDF2) expedited degradation to a normal level compared with hypoxia treatment only (Hyp‐LV‐NC). As shown in Figures 4B and C, the half‐life of EZH2 mRNA was shortened in YTHDF2‐overexpressing cells under hypoxia and prolonged in YTHDF2‐silenced human primary ESCs under normoxia.

The m^6^AVar database^54^ indicated the presence of very high confidence m^6^A modification motifs in EZH2 mRNA (Figure 3G), and this motif was used to construct the mutant in Dual‐luciferase reporter assay. As shown in Figure 4D, silencing YTHDF2 in THESC cells increased WT reporter luciferase activity (the left column of Figure 4D), but had no effect on mutant construct luciferase activity (the right column of Figure 4D). These results demonstrate that YTHDF2 can directly bind to m^6^A modification sites to promote EZH2 mRNA degradation in human ESCs.

Furthermore, no specific m^6^A peak was found in the mRNAs of decidua markers IGFBP1 and PRL via IGV analysis (Figure S3A), ruling out the potential direct regulation of m6A on these molecules. Figure S3B shows the significantly changed genes and differentially enriched KEGG pathways in the hypoxia group. Specifically, the hypoxia group had 586 up‐regulated genes compared with the normoxia group (left column of Figure S3B). The HIF‐1α signalling pathway, oxidative phosphorylation signalling and glycolysis/gluconeogenesis signalling, which are associated with hypoxia, were all enriched in the hypoxia group (right column of Figure S3B).

### 3.7 EM patient SE shows increased ALKBH5 and decreased YTHDF2 protein expression levels

Nuclear staining of ALKHB5 and cytoplasmic staining of YTHDF2 were observed in human ESCs (Figure 4E). ALKBH5 expression levels were increased in ESCs from EM patient SE compared with levels in ESCs from control patient SE, while YTHDF2 expression levels were decreased in ESCs from EM patient SE compared with levels in ESCs from control patient SE (Figure 4E). The ALKBH5 and YTHDF2 *H*‐scores in ESCs are summarised in Figure 4F. IF staining for phalloidine showed that no abnormal morphology of human primary ESCs from EM patients was observed after ALKBH5 silencing under hypoxia and MPA + cAMP combined treatment, suggesting ALKBH5‐dependent decidualization damage under hypoxia (Figure 4G).

### 3.8 ALKBH5 silencing can rescue hypoxia‐inhibited decidualization by increasing m^6^A modification of EZH2 mRNA and decreasing EZH2 protein expression levels

**FIGURE 5:**
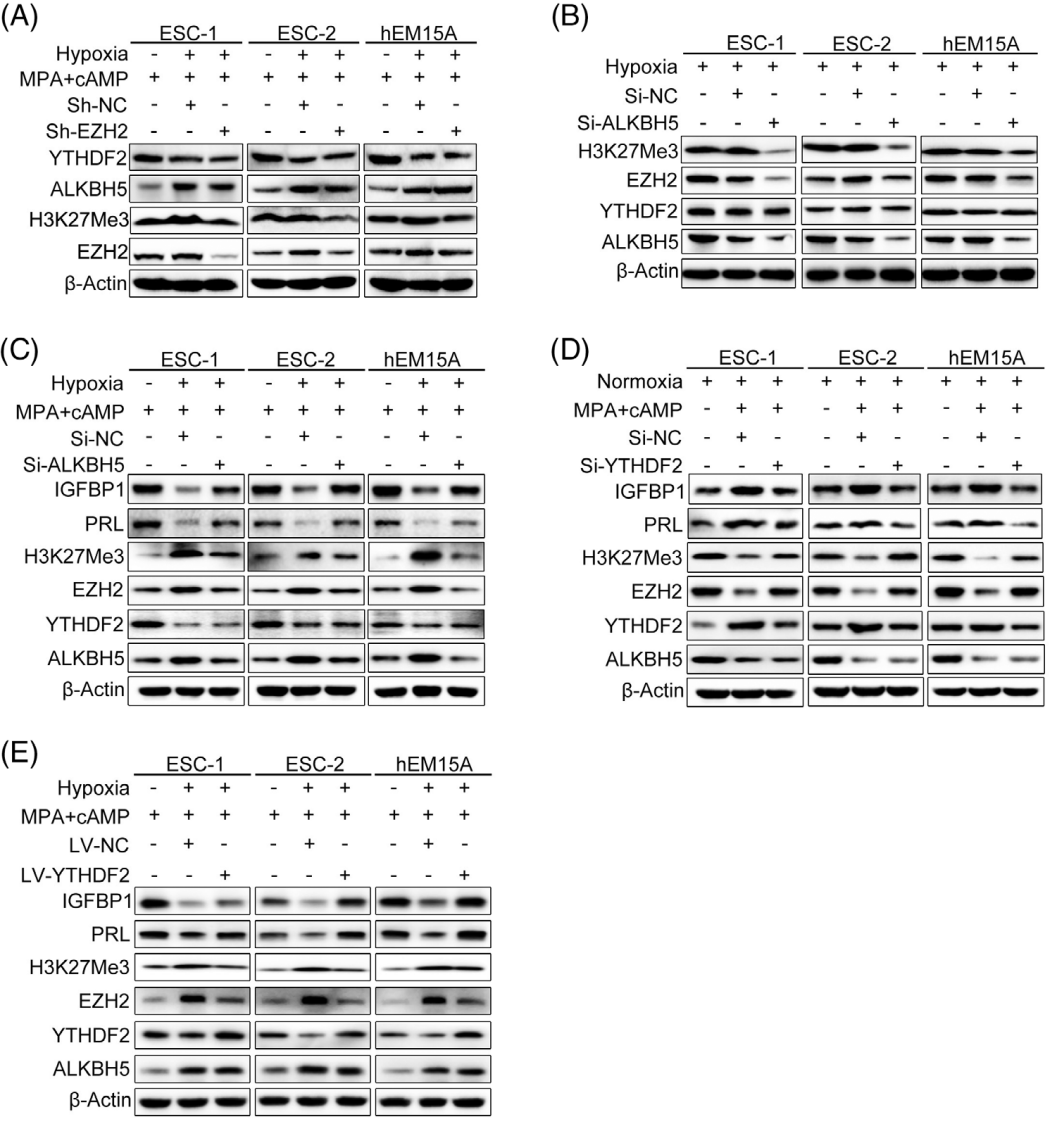
Hypoxia inhibited decidualization by increasing m^6^A modification of EZH2 mRNA in an ALKBH5‐ and YTHDF2‐based manner. (A) Western blot analysis of EZH2, H3K27Me3, ALKBH5 and YTHDF2 protein levels in human primary ESCs and hEM15A cells after treatment with MPA + cAMP for 72 h, hypoxia and MPA + cAMP combined treatment for 72 h, or Sh‐EZH2 for 48 h followed by hypoxia and MPA + cAMP combined treatment for 72 h. (B) Western blot analysis of ALKBH5, YTHDF2, EZH2 and H3K27Me3 protein levels in human primary ESCs and hEM15A cells after treatment with Si‐ALKBH5 or Si‐NC for 48 h under hypoxia. (C) Western blot analysis of ALKBH5, YTHDF2, EZH2, H3K27Me3, PRL and IGFBP1 protein levels in human primary ESCs and hEM15A cells after treatment with MPA + cAMP for 72 h, hypoxia and MPA + cAMP combined treatment for 72 h, or Si‐ALKBH5 for 48 h followed by hypoxia and MPA + cAMP combined treatment for 72 h. (D) Western blot analysis of ALKBH5, YTHDF2, EZH2, H3K27Me3, PRL and IGFBP1 protein levels in human primary ESCs and hEM15A cells after treatment with MPA + cAMP for 72 h or Si‐YTHDF2 for 48 h followed by MPA + cAMP combined treatment for 72 h. (E) Western blot analysis of ALKBH5, YTHDF2, EZH2, H3K27Me3, PRL and IGFBP1 protein levels in human primary ESCs and hEM15A cells after treatment with MPA + cAMP for 72 h, hypoxia and MPA + cAMP combined treatment for 72 h, or LV‐YTHDF2 for 48 h followed by hypoxia and MPA + cAMP combined treatment for 72 h.

We next explored the relationship between histone methylation and m^6^A methylation modifications under hypoxia or decidualization. Hypoxia increased EZH2 and ALKBH5 expression and decreased YTHDF2 protein expression with decidualization induction, while EZH2 knockdown did not influence ALKBH5 or YTHDF2 expression (Figure 5A). These results indicate that increased histone methylation modification with hypoxia does not interfere with m^6^A methylation modification, but decreased m^6^A methylation modification can increase EZH2 expression levels. Silencing ALKBH5 under hypoxic conditions can decrease EZH2 and H3K27Me3 protein expression, but did not affect YTHDF2 expression (Figure 5B). Additionally, knockdown of ALKBH5 under hypoxia and MPA + cAMP combined treatment reduced EZH2 and H3K27Me3 protein expression and rescued the decreased IGFBP1 and PRL expression suppressed by hypoxia (Figure 5C). To further elucidate the role of YTHDF2 in decidualization, YTHDF2 was silenced in human ESCs (Figure S2A). YTHDF2 knockdown under normoxia and MPA + cAMP combined treatment increased EZH2 and H3K27Me3 protein expression levels compared with decidualization induction only (Figure 5D). It also decreased IGFBP1 and PRL expression, but did not influence ALKBH5 protein expression levels (Figure 5D). However, overexpression of YTHDF2 under hypoxia and MPA + cAMP combined treatment reduced EZH2 and H3K27Me3 expression compared with hypoxia and MPA + cAMP combined treatment only (Figure 5E). Furthermore, it increased IGFBP1 and PRL expression, with no effect on ALKBH5 protein expression (Figure 5E).

### 3.9 Selective Ezh2 depletion in mouse ESCs increases endometrial receptivity and improves mouse fertility

**FIGURE 6:**
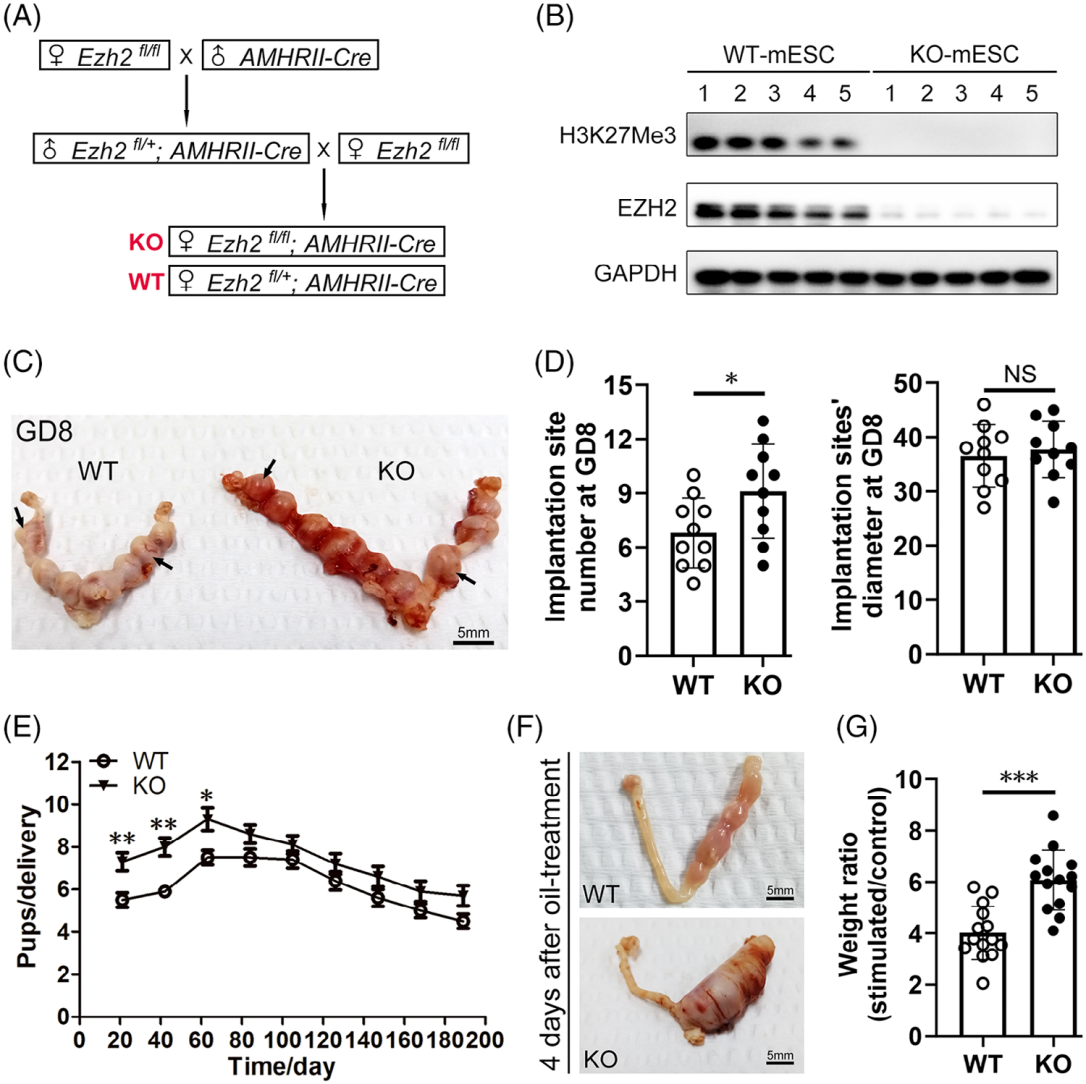
Selective *Ezh2* depletion in mouse ESCs increases endometrial receptivity and improves female mouse fertility. (A) Schematic showing the generation of ESC‐specific knockout of *Ezh2* mice. *Ezh2* ^fl/fl^; *AMHRII‐Cre* (KO) mice were obtained by mating *Ezh2* ^fl/+^; *AMHRII‐Cre* mice (WT mouse) with *Ezh2* ^fl/fl^ mice. (B) Western blot analysis of EZH2 and H3K27Me3 protein levels in fresh extracted ESCs from 6‐week‐old paired WT or KO mouse uteri (*n* = 5 for WT group, *n* = 5 for KO group). (C) Representative picture showing the gross blastocyst morphology of 6‐week‐old WT or KO mice at GD8 (day 8 after embryo implantation). The black arrowheads point to an example implantation site. Scale bars = 5 mm. (D) Histogram showing the difference of implantation site number and implantation site diameter between KO and WT mice at GD8 (*n* = 10 for WT group, *n* = 10 for KO group). Paired *t*‐test. (E) The development of mouse fertility within 6 months of mating. The horizontal axis shows time (days) from mating and the vertical axis indicates average pup number of each delivery (*n* = 10 for WT group, *n* = 10 for KO group). (F) Representative pictures showing the gross morphology of oil‐treated uterine horns and untreated horns from WT and KO mice collected 4 days after oil injection. Scale bars = 5 mm. (G) The ratio of the wet weight of oil‐treated horn to the wet weight of untreated horn calculated from 14 WT mice and 14 KO mice at day 4 after oil injection. Unpaired *t*‐test.

To explore the function of *Ezh2* on decidualization, we generated ESC‐specific *Ezh2* KO mice (KO mouse: *Ezh2* ^fl/fl^; *AMHRII*‐*Cre*) (Figure 6A). The oestrous cycle, number of offspring and body weight of pups were normal in *Ezh2* ^flox/+^; *AMHRII‐Cre* females and *Ezh2* ^flox/+^; *AMHRII‐Cre* males. In other words, *Ezh2* ^flox/+^; *AMHRII‐Cre* mice displayed normal fertility. The successful disruption of EZH2 led to markedly decreased H3K27Me3 protein levels in ESCs from 6‐week‐old mice (Figure 6B). The number of implantation sites visible in WT (*n* = 10) and KO females (*n* = 10) was counted. Representative images in Figure 6C show the gross morphology of blastocysts in uterine horns. We observed increased numbers of implantation sites in KO females at GD8 (the left column of Figure 6D), but no difference in the diameter of implantation sites at GD8 (the right column of Figure 6D). Additionally, no abnormal growth or body weight changes of the offspring were observed in the later period (0 to 6 weeks old). Fertility tests demonstrated the increased fertility of KO mice (Figure 6E). No significant differences in body weight or 6‐week‐old mouse ovary weight were found between KO group and WT females (Figures 3C and D). There were nine gravidities seen in KO mice within 6 months, which was similar to the number of gravidities in WT mice (Figure 6E). There were eight deliveries within 6 months for both KO and WT mice (Figure 6E). Both KO and WT mice need a 21 days to produce new pups (Figure 6E). KO mice had an increased average number of pups per delivery in the first three deliveries, but no significant increase was observed after the third delivery. We hypothesised that this phenomenon is possibly related to the increased mouse age, but the detailed mechanism needs further research. KO mice had a sensitive response to decidual stimuli (Figure 6F), characterised by dramatically increased wet weight of the oil‐treated horn (Figure 6G).

Anti‐Müllerian hormone type 2 receptor (AMHRII) is primary expressed in the Müllerian duct mesenchyme and slightly expressed in the ovarian granulosa cells.^55^, ^56^, ^57^ Therefore, to rule out the effects of *Ezh2* KO on the ovary, we examined the antral follicle number, cumulus–oocyte complex (COC) expansion rate, and corpora lutea number in WT or KO mice. No significant difference was found in antral follicle counts per ovary after PMSG for 48 h (Figure S3E), which implies comparable follicular development in WT and KO mice. The COC expansion rate was similar between KO mice and WT mice, even with 100 ng/mL FSH treatment for 12 h (Figure S3F). This indicates that the increased female fecundity in KO mice was not caused by increased COC expansion. No significant difference was found in corpora lutea counts per ovary between KO and WT mice at 16 h post‐HCG injection (Figure S3G). Serum oestrogen and progesterone concentrations were also similar between 6‐week‐old KO and WT mice (Figure S3H), revealing that specific *Ezh2* KO via *AMHRII*‐Cre did not influence the endocrine function of the mouse ovary.

### 3.10 Selective Ezh2 depletion in mouse ESCs contributes to decidualization by increasing IGFBP1 protein expression levels

Epigenetic modifications can change dramatically during decidualization. While the expression levels of HIF‐1α, EZH2 and H3K27Me3 in SE ESCs from EM patients were markedly higher than those in the Con group, the expression patterns of these proteins were similar in SE ESCs from Con and EM patients (Figure S4A). IHC results showed that HIF‐1α protein expression in ESCs gradually increased from the early proliferative phase of the menstrual cycle (D7–D10), reached the maximum level at the terminal proliferative phase of the cycle (D11–D14) and then gradually decreased in secretory phase (D15–D25). The lowest HIF‐1α levels were observed in the terminal secretory stage of the menstrual cycle (D20–D25), also known as the window period (7−10 days after ovulation) for embryo implantation. EZH2 and H3K27Me3 protein expression levels in ESCs gradually increased from D7 to D10, reached the maximum level at D15–D19 and then dramatically decreased during the window period for embryo implantation (D20–D25). These results indicate the importance of coordinated histone methylation modification during decidualization.

**FIGURE 7:**
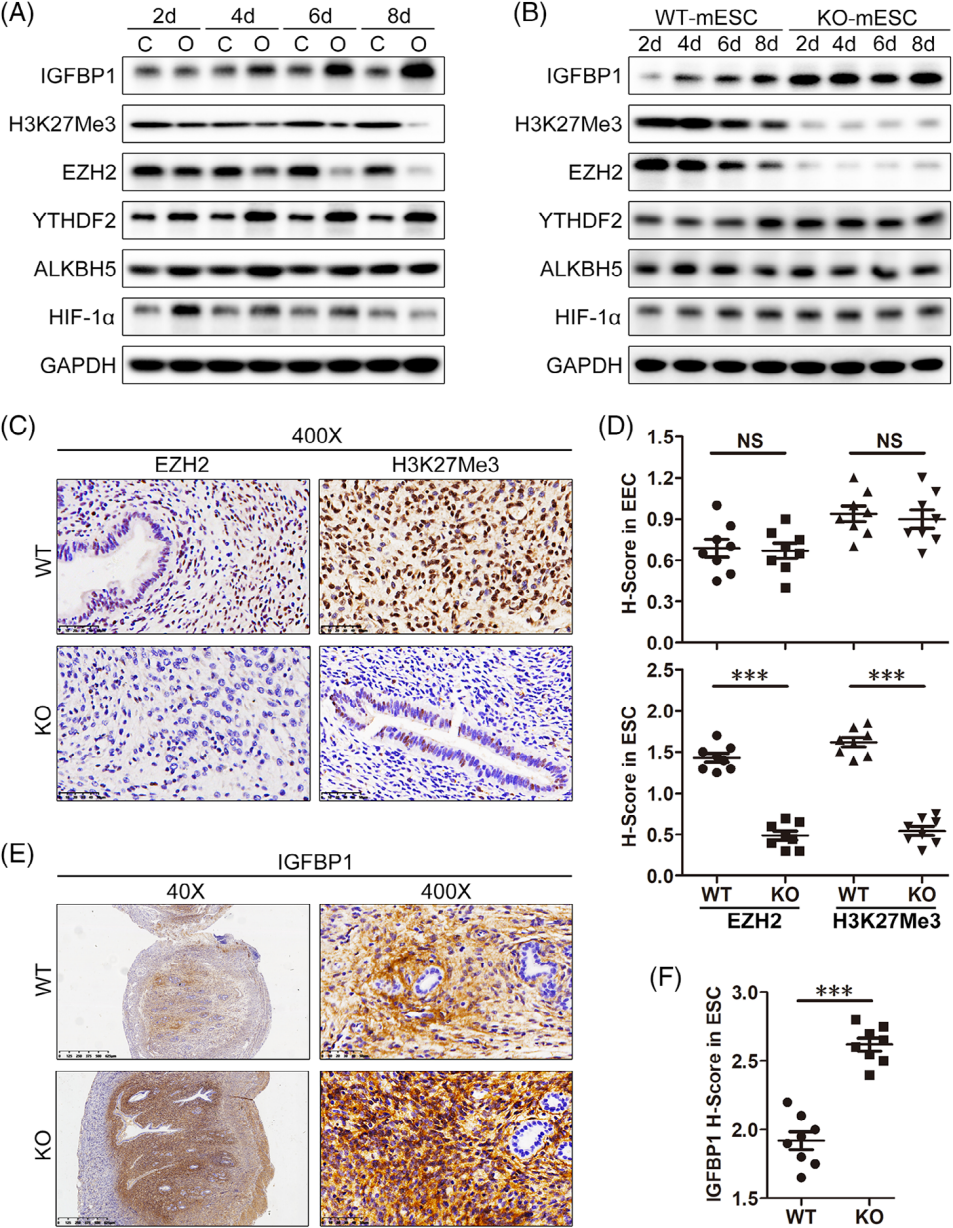
Selective *Ezh2* depletion in mouse ESCs contributes to decidualization by increasing IGFBP1 protein expression. (A) Western blot analysis of HIF‐1α, ALKBH5, YTHDF2, EZH2, H3K27Me3 and IGFBP1 protein levels in normal mouse ESCs (mESCs) after inducing in vivo decidualization for 2, 4, 6 or 8 days. (B) Western blot analysis of HIF‐1α, ALKBH5, YTHDF2, EZH2, H3K27Me3 and IGFBP1 protein levels in WT or KO mouse ESCs after inducing in vivo decidualization for 2, 4, 6 or 8 days. (C) IHC photomicrographs of EZH2 and H3K27Me3 staining in mESCs from 6‐week‐old WT or KO mice. Scale bars = 50 μm, original magnification: ×400. (D) The summarised *H*‐scores of EZH2 and H3K27Me3 in EECs and ESCs from WT or KO mice (*n* = 8 for WT group, *n* = 8 for KO group). Paired *t*‐test. (E) IHC photomicrographs of IGFBP1 staining in mESCs from 6‐week‐old WT or KO mice. Scale bars = 625 or 50 μm, original magnification: ×40 or ×400. (F) *H*‐score of IGFBP1 in ESCs from WT or KO mice (*n* = 8 for WT group, *n* = 8 for KO group). Paired *t*‐test.

We established artificially induced decidualization mouse models in vivo to explore the roles of specific proteins in decidualization. Western blot was used to explore the protein expression patterns of HIF‐1α, ALKBH5, YTHDF2, EZH2, H3K27Me3 and IGFBP1 during in vivo decidualization (Figure 7A). Our results show that HIF‐1α protein expression reached the highest level 2 days after oil was injected into the uterine lumen, then gradually decreased until 8 days after oil injection. A significant induction of ALKBH5 was noted at 4 days after oil injection, but a sharply decrease was then seen at 6 days and 8 days after oil injection. Figure 5E shows that MPA + cAMP treatment (in vitro decidualization) could suppress ALKBH5 expression compared with normoxia, which seems inconsistent with the in vivo data. However, hypoxia/HIF‐1α is a real event during in vivo decidualization (Figure 7A), making it is possible that the in vitro data are not consistent with the in vivo data. These results also demonstrate the importance of hypoxia in decidualization. YTHDF2 protein expression levels rapidly increased after oil injection, with the maximal level achieved at 4 days after oil injection. The levels remained stable until 8 days after oil injection. EZH2 and H3K27Me3 protein levels gradually decreased after oil injection and were lowest at 8 days after oil injection. Conversely, IGFBP1 protein levels gradually increased after oil injection and peaked at 8 days after oil injection. The expression patterns of HIF‐1α, ALKBH5, YTHDF2, EZH2, H3K27Me3 and IGFBP1 during in vivo decidualization are summarised in Figure S4B.

KO mice showed markedly reduced EZH2 and H3K27Me3 and notably increased IGFBP1 protein expression levels in ESCs during in vivo decidualization (Figure 7B). However, specific EZH2 KO in mouse ESCs did not influence the expression pattern of HIF‐1α, ALKBH5 or YTHDF2 during in vivo decidualization (Figure 7B), consistent with our in vitro results (Figure 5A). IHC results demonstrated the selective deficiency of EZH2 in ESCs from KO mice (Figure 7C), but the decidua marker IGFBP1 was dramatically expressed in ESCs from KO mice compared with that in ESCs from WT mice at 4 days after oil infusion (Figures 7E and F). At the same magnification, the KO mice decidual uterus volume was significantly larger than that of WT mice, suggesting that KO mice had a sensitive response to decidual stimuli in vivo (Figure 7E). The *H*‐scores of mouse EECs or mouse ESCs are summarised in Figures 7D and F, respectively. No differences in EZH2 protein and H3K27Me3 protein were found in mouse EECs.

### 3.11 Decidua from EM model mice show increased HIF‐1α, ALKBH5, EZH2 and H3K27Me3 expression and decreased YTHDF2 and IGFBP1 expression

**FIGURE 8:**
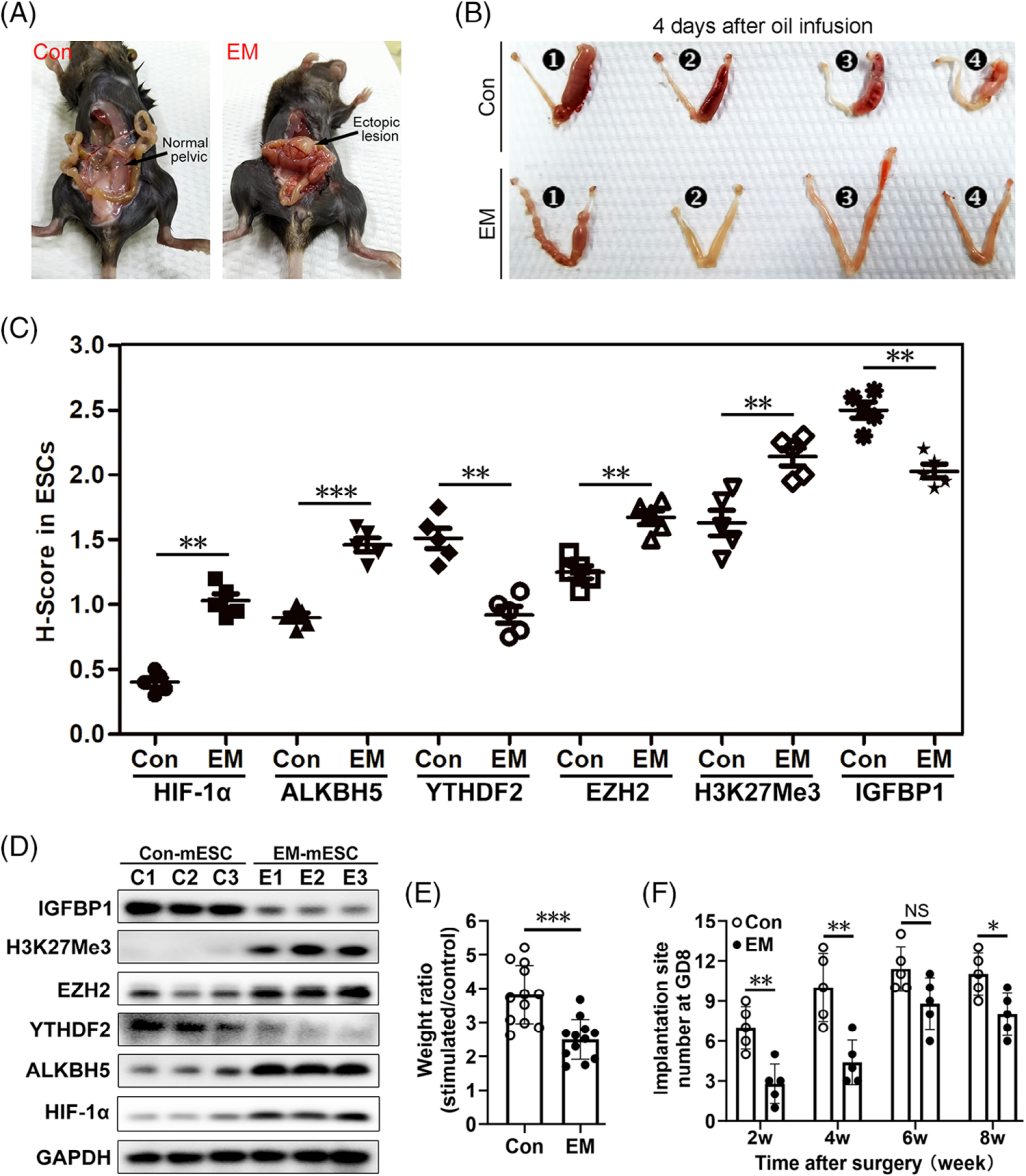
Decidua from endometriosis mice model show increased HIF‐1α, ALKBH5, EZH2 and H3K27Me3 expression, but decreased YTHDF2 and IGFBP1 expression. (A) Representative visible lesions within the peritoneal cavity of EM mice. No ectopic adhesion lesions or inflammation were observed in the Con group. (B) Representative image of oil‐treated uterine horns and untreated horns from Con and EM mice collected 4 days after oil injection. (C) Summarised *H*‐scores of HIF‐1α, ALKBH5, YTHDF2, EZH2, H3K27Me3 and IGFBP1 in ESCs from Con or EM mice (*n* = 5 for Con group, *n* = 5 for EM group). Paired *t*‐test. (D) Western blot analysis of HIF‐1α, ALKBH5, YTHDF2, EZH2, H3K27Me3 and IGFBP1 protein levels in fresh extracted mouse ESCs. Fresh ESCs were extracted from Con or EM mouse uteri after oil injection for 4 days. C1–C3 and E2–E3 represent fresh ESC samples from Con or EM mice, respectively. Each sample contained ESCs mixed from three mice. (E) The ratio of the wet weight of oil‐treated horn to the wet weight of untreated horn calculated from 12 Con mice and 12 EM mice at day 4 after oil injection. Unpaired *t*‐test. (F) Histogram showing the implantation site number in Con and EM mice at GD8 (*n* = 5 for Con group at each time point, *n* = 5 for EM group at each time point). 2w, 4w, 6w and 8w indicate the weeks after endometrium transplantation surgery. Paired *t*‐test.

EM mouse model were established using C57BL/6 mice as previously described.^32^ The detailed protocol is shown in Figure S4C. Figure 8A shows images of typical EM cysts. Two weeks after the endometrium transplantation surgery, Con and EM mice were mated with fertile males to induce pregnancy, or mated with vasectomised males to induce pseudopregnancy. Our results indicate impaired decidualization of EM mice at 4 days after oil infusion (Figure 8B). Four days after oil infusion, the uteri were collected for IHC analysis, and the wet weights of the uterine horns were recorded. IHC revealed increased HIF‐1α, ALKBH5, EZH2 and H3K27Me3 protein expression levels in ESCs from EM mice at 4 days after oil infusion (Figure 8C), while the expressions levels of YTHDF2 and IGFBP1 were reduced in EM mice compared with levels in the Con mice (Figure 8C). These findings were consistent with our western blot results (Figure 8D). The ratio of the wet weight of the oil‐treated horn to the wet weight of the untreated horn was decreased in the EM group compared with that in the Con group (Figure 8E). Previous studies have reported decreased fertility in EM mice.^32^ Our current results also suggest markedly decreased implantation sites in EM females, but no difference was observed at 6 weeks after endometrium transplantation (Figure 8F). We did not observe any abnormal growth or body weight changes in the EM offspring in the later period (0–6 weeks old).

## 4 DISCUSSION

In summary, we illustrate in this article that hypoxia increased the expression of demethylase ALKBH5 and decreased the expression of reader protein YTHDF2, which work together to up‐regulated EZH2 protein expression via decreasing the m6A modification of EZH2 mRNA. Increased EZH2 and H3K27Me3 expression in ESCs finally contributes to defective decidualization of EM by decreasing the expression of decidua marker IGFBP1. Histone methyltransferase EZH2 and its well‐known repressive mark H3K27Me3 plays an important role in the aetiology of EM.^30^, ^31^ EZH2 and H3K27Me3 were highly expressed in the heterotopic endometrial lesions of EM patients compared with those in eutopic and control endometria.^29^, ^58^, ^59^, ^60^ Moreover, we demonstrated in previous research that decreased EZH2 expression and reduced H3K27Me3 levels in EM ovarian granulosa cells can disturb ovulation and lead to EM‐associated infertility.^61^ However, no study has explored the roles of EZH2 in EM decidualization. Decidualization failure is associated with a disturbance of oestrogen and progesterone, abnormal inflammation, imbalanced expression of multiple endocrine, paracrine and autocrine modulators in the endometrium, epigenetic modifiers and chromatin modifications.^6^ Here, we show for the first time that increased EZH2 and H3K27Me3 levels in the eutopic endometrium of EM impair decidualization through down‐regulation of IGFBP1 expression through its proximal promoter. Therefore, controlling the expression of EZH2 in the eutopic endometrium of EM may be therapeutically beneficial in the future. In addition, IGFBP1 is not only a marker of decidualization, but also has important significance in the diagnosis of EM. Based on single‐cell analysis results, menstrual shed endometrium from EM has less IGFBP1 positive decidualised ESCs when compared with controls, which also confirming compromised decidualization of EM.^62^, ^63^ Labelling IGFBP1 of menstrual effluent may provide an effective, non‐invasive diagnostics tool for identifying EM from patients with corresponding symptoms.^62^

Poor endometrial receptivity is associated with local hypoxic stress,^19^, ^64^ and HIF‑1α is activated by hypoxia and elevated in the peri‐implantation endometrium of women with recurrent miscarriage.^65^ Hypoxia is a common stress that can evoke substantial post‐transcriptional and translational regulation in cells. Total mRNA m^6^A levels were reduced during hypoxia, which possibly results from an altered balance between m^6^A writers and erasers. To explore the influence of hypoxia on m^6^A levels in ESCs, we examined the protein expression levels of different writers, erasers and readers. ALKBH5 was a significantly changed eraser protein in ESCs, YTHDF2 was systematically down‐regulated under hypoxia, and no changes were observed in METTL3, WTAP or FTO. Interestingly, ALKBH5, a primary m^6^A demethylase, not only plays a key role in human reproductive system diseases,^49^ but is also believed to be directly regulated by HIF‑1α under hypoxia.^23^, ^49^, ^50^, ^51^ YTHDF2, a common m^6^A reader, is suppressed by hypoxia and selectively recognises m^6^A‐modified mRNAs, resulting in their degradation.^66^ Consistent with previous evidence,^24^, ^67^, ^68^ our data suggest that YTHDF2 expression is also associated with hypoxia. But the effect of hypoxia on gene expression regulation is very complex, so more studies are needed to verify if HIF‑1α can directly suppress YTHDF2 levels.

We explored the cause of increased EZH2 expression in the eutopic endometrium of EM and found m^6^A methylation modification of EZH2 transcription to be an important reason for the impaired decidualization. Although the regulatory mechanisms of m^6^A methylation and histone methylation in ESCs are complex, EZH2 is a critical linker between the two processes. M^6^A RNA methylation is an abundant nucleotide modification in mRNAs, but our data are the first to demonstrate that it plays a role in EM decidualization. Analysis of clinical samples showed that ALKBH5, YTHDF2 and EZH2 levels were strongly correlated with EM. In mouse models, EZH2 depletion enhanced decidualization by increasing IGFBP1 expression levels. However, inhibiting EZH2 had no effects on the expression levels of writers, erasers or reader proteins. Collectively, our work reveals a vital function for ALKBH5‐mediated m^6^A methylation in the hypoxic microenvironment of EM and identifies EZH2 as a crucial target of m^6^A modification in the decidualization of ESCs. M^6^A RNA methylation and histone methylation modification processes all involve the regulation of multiple molecules, and a growing number of new mechanisms are being proposed and investigated. Our study demonstrates that the hypoxia/ALKBH5‐YTHDF2/EZH2/IGFBP1 axis acts as a critical regulator in decidualization of EM, linking the hypoxic microenvironment and histone methylation to decidualization.

Two recently studies showed that adult female mice with a conditional deletion of *Ezh2* in both epithelial and stromal cell of uterus result in pregnancy loss via disturbing epithelial and stromal differentiation.^69^, ^70^ But as the authors described in their introduction, successfully implantation depends on a series of ordered events involving embryo attachment, epithelial proliferation, stromal cell differentiation and embryo invasion. Epithelial and stromal cells do not change together during decidualization, which means the EZH2–PRC2–H3K27me3 axis may play different roles in epithelial and stromal cells, and these two types of cells also influence each other during decidualization. We just focused on the influence of EZH2 in ESCs via conditional deletion of *Ezh2* in stromal cells via *AMHRII‐Cre* but ont *Pgr‐Cre*. Although many efforts have been made to determine the specific epigenetic modifications of ESCs during decidualization, the pathologic changes of EECs have not been examined alone. According to our IHC results of human endometrium tissue, EZH2 was moderately expressed in the nuclei of human ESCs, but positively expressed in both the nuclei and cytoplasm of human EECs. Histone methyltransferase EZH2 can be expressed in the nucleus or cytoplasm of cells, and the cytoplasmic accumulation of EZH2 in EECs suggests a possible change in function. However, no solid conclusions can be made at present. Successful embryo implantation requires the interaction between EECs and the underlying ESCs. Therefore, more studies on EECs are required to better understand the molecular details of decidualization. As another important decidual marker, PRL protein expression also changed following EZH2 inhibition, but no histone methylation modification site was found in the PRL gene promoter. The increased protein expression levels of PRL after EZH2 inhibition may be caused by other endocrine, paracrine or protein interactions.

## AUTHOR CONTRIBUTIONS

Xiang Lin and Weijia Gu performed the experiments. Xiang Lin, Weijia Gu, Yi Zhang, Feng Zhuo, Fanxuan Zhao, Xiaoying Jin, Chao Li and Dong Huang collected the clinical samples. Xiang Lin and Yongdong Dai analysed the data, made the figures and drafted the article. Yongdong Dai, Songying Zhang, Dong Huang and Chao Li critically reviewed the article. Xiang Lin and Songying Zhang designed and conceived the project. Songying Zhang supervised the study and was responsible for critical revision of the manuscript for important intellectual content.

## CONFLICT OF INTEREST STATEMENT

The authors declare that they have no conflict of interest.

## ETHICS STATEMENT

This study was started on 1 January 2020 and finished on 30 August 2022. This study was monitored by the ethics committee of Sir Run Shaw Hospital, Zhejiang University (approval number: 20211019050815690). Informed written consent was obtained from all patients before sample collection. Mice were bred in accordance with the National Institutes of Health Guide for the Care and Use of Laboratory Animals.

## Supporting information

Figures S1‐S4

Figure S1

Figure S2

Figure S3

Figure S4

Figure S5

Supporting Information

## Data Availability

The sequencing data were uploaded in the GEO database and will be available with manuscript publication. The original contributions presented in the study are included in the article or supplemental materials. Further inquiries can be directed to the corresponding author.
