# Supplementary material for: The involvement of RNA N6‐methyladenosine and histone methylation modification in decidualization and endometriosis‐associated infertility
Source: Clin Transl Med. 2024 Feb 12;14(2):e1564. doi: 10.1002/ctm2.1564 (PMC10859880; doi:10.1002/ctm2.1564)
Supplement: Supplementary file 1 — Figures S1‐S4 [file CTM2-14-e1564-s003.docx]

**Supplemental Figure 1:**

**A.** IGV was used to visualize the ChIP-sequencing results. The input represents reads from chromatin pellets before immunoprecipitation and IP means enriched reads after immunoprecipitation with the H3K27Me3 antibody. **B.** ChIP-sequencing was performed in three different human primary ESCs with EZH2 knockdown (Sh-EZH2) or negative control treatment (Sh-NC). KEGG pathways that were differentially enriched in the Sh-EZH2 group are listed in the bubble diagram, and the right panel shows the Protein-Protein Interaction networks of five differentially enriched KEGG pathways. **C.** IHC photomicrographs of HIF-1α protein expression in SE from Con and EM patients. Scale bars = 50 µm, original magnification: ×100 or ×400. The right panel shows the summarized H-scores of HIF-1α in ESCs of SE from Con and EM patients (n=28 for Con group, and n=64 for EM group). Unpaired t-test. **D.** IF analysis of human primary ESCs showing the nuclear localization of EZH2, increased expression levels of EZH2 after hypoxic treatment, and reduced expression levels of EZH2 after decidualization induction. DAPI (blue) was used to stain cellular nuclei. Original magnification: ×400. **E.** Western blot analysis showing protein expression of KDM6A, EZH2, H3K27Me3, H3K4Me3, H3K9Me3, H3K36Me3, and H3K79Me3 in human primary ESCs treated with normoxia (Con), MPA+cAMP (Dec), or hypoxia (Hyp) for 72 hours. **F.** Flow cytometry analysis showing the cell cycle distribution of eight different human primary ESCs after Con, Hyp, Dec, or DH treatment for 72 hours. Each value represents the mean ± standard deviation of three independent experiments. Paired t-test. **G.** Flow cytometry analysis showing the percentage of apoptotic human primary ESCs after Con, Hyp, Dec, or DH treatment for 72 hours (n=6 for human primary ESCs). Paired t-test.

**Supplemental Figure 2:**

**A.** Western blot analysis was used to confirm the infection efficiency of Sh-HIF-1α (1#, 2#, 3#) and Sh-EZH2 (1#, 2#, 3#), and the knockdown efficiency of Si-ALKBH5 (1#, 2#, 3#) and Si-YTHDF2 (1#, 2#, 3#). NC represents the negative control, and 1# – 3# represents the different Sh/Si-RNAs used to target the same gene. The red mark indicates the final used item. **B.** The peak density of m^6^A distribution. **C.** Detailed information of four HIF-1α binding sites in the ALKBH5 promoter, as predicted using the JASPAR database. **D.** qRT-PCR results of HIF-1α, ALKBH5, and YTHDF2 mRNA expression in human primary ESCs under normoxic or hypoxic conditions for 24 hours. Paired t-test. **E.** qRT-PCR results of HIF-1α, ALKBH5, and YTHDF2 mRNA expression in human primary ESCs treated with Sh-HIF-1α or Sh-NC under hypoxia for 48 hours. Paired t-test. **F.** Western blot analysis of HIF-1α, ALKBH5, and YTHDF2 protein levels in human primary ESCs after treatment with Sh-HIF-1α or Sh-NC under hypoxia for 72 hours. **G.** qRT-PCR results of HIF-1α, ALKBH5, and EZH2 mRNA expression in human primary ESCs after transfection with Si-ALKBH5 or Si-NC under hypoxia for 48 hours. Paired t-test.

**Supplemental Figure 3:**

**A.** IGV was used to visualize the MeRIP-sequencing results of human primary ESCs treated with normoxia or hypoxia for 48 hours. No reads were found in the promoters of decidua marker genes IGFBP1 and PRL after hypoxic culture for 48 hours. **B.** After normoxia or hypoxia treatment for 48 hours, MeRIP-sequencing was performed in human primary ESCs. The left volcano plot shows the significantly upregulated and downregulated genes after hypoxia culture, and the right bubble diagram displays the differentially enriched KEGG pathways after hypoxia culture. **C.** The body weight of pups produced by 8-week-old WT or KO mice (n=15 for WT group, n=15 for KO group). Newborn, 3 weeks, 6 weeks, and 8 weeks refers to the age of pups. Unpaired t-test. **D.** The ovary weight of 6-week-old WT or KO mice (n=15 for WT group, n=15 for KO group). Unpaired t-test. **E.** The number of antral follicles per ovary after PMSG treatment for 48 hours (P48) were counted by two technicians (n=15 for WT group, n=15 for KO group). Unpaired t-test. **F.** Cumulus-oocyte complexes (COC) were acquired after culture with 100 ng/mL FSH for 12 hours. Histogram showing the statistical analysis result of the average COC expression rate from 10 WT or paired 10 KO mice. Paired t-test. **G.** Histogram showing the corpora lutea counts per ovary from 15 WT or 15 KO mice at 16 hours post-HCG injection (H16). Unpaired t-test. **H.** The left panel shows ELISA results of serum estrogen (E2) concentrations in 6-week-old WT or KO mice at P48 (n=14 for WT group, n=14 for KO group). Unpaired t-test. The right panel shows ELISA results of serum progesterone concentrations in 6-weeks-old WT or KO mice at H4 (n=15 for WT group, n=15 for KO group). Unpaired t-test. H4 refers to 4 hours post-HCG injection.

**Supplemental Figure 4:**

**A.** Summarized H-scores of HIF-1α, EZH2, and H3K27Me3 in ESCs of SE from Con and EM patients. D7–D10 means the endometrium was collected from day 7 to day 10 of the menstrual cycle from Con or EM patients. D11–D14 means the endometrium was collected from day 11 to day 14 of the menstrual cycle from Con or EM patients. D15–D19 means the endometrium was collected from day 15 to day 19 of the menstrual cycle from Con or EM patients. D20–D25 means the endometrium was collected from day 20 to day 25 of the menstrual cycle from Con or EM patients. n=11 for Con and n=8 for EM at D7–D10, n=13 for Con and n=32 for EM at D11–D14, n=16 for Con and n=45 for EM at D15–D19, n=12 for Con and n=19 for EM at D20–D25. Unpaired t-test or Mann Whitney test. **B.** The protein expression levels of HIF-1α, ALKBH5, YTHDF2, EZH2, H3K27Me3, and IGFBP1 shown in Figure 7A were quantified using Image J software. GAPDH was used as an internal control. * *P*<0.05, 4d *vs* 2d; # *P*<0.05, 6d *vs* 2d; & *P*<0.05, 8d *vs* 2d. **C.** Detailed flow charts of the establishment of the EM mouse model and animal experiments.
