# Supplementary figures and images for: The involvement of RNA N6‐methyladenosine and histone methylation modification in decidualization and endometriosis‐associated infertility
Source: Clin Transl Med. 2024 Feb 12;14(2):e1564. doi: 10.1002/ctm2.1564 (PMC10859880; doi:10.1002/ctm2.1564)

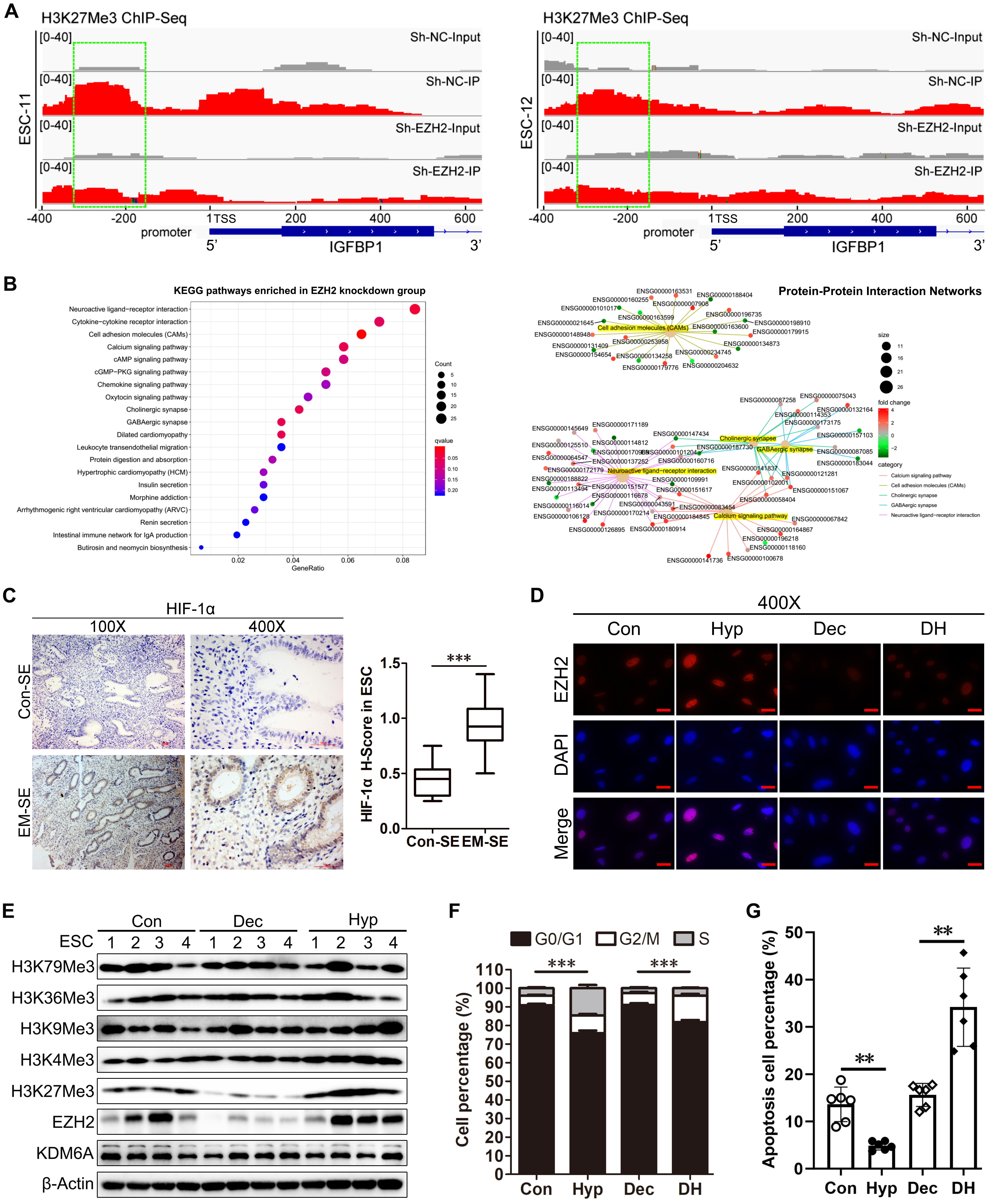

Supplement: Supplementary file 2 — Figure S1 [file CTM2-14-e1564-s005.jpg]

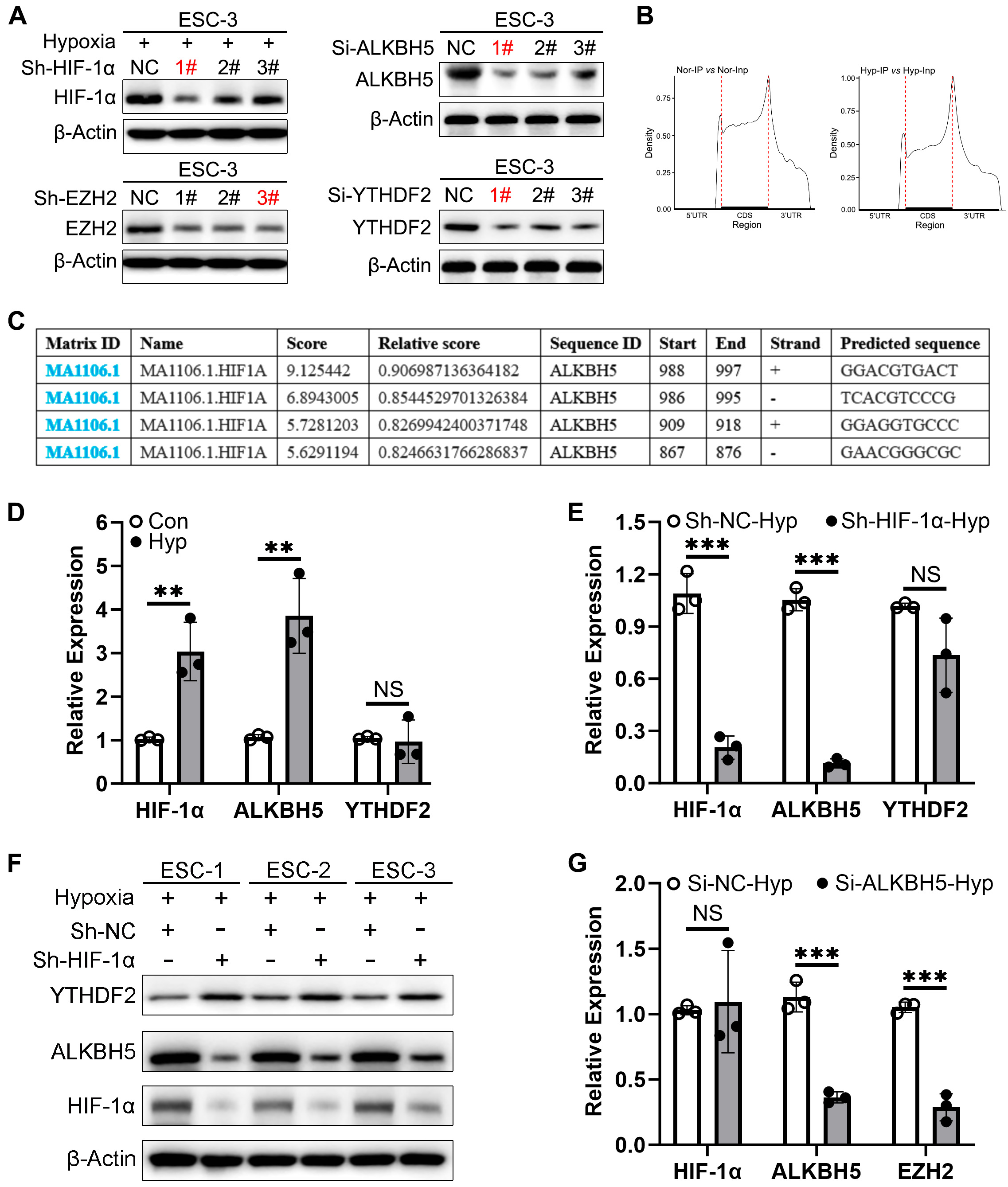

Supplement: Supplementary file 3 — Figure S2 [file CTM2-14-e1564-s007.jpg]

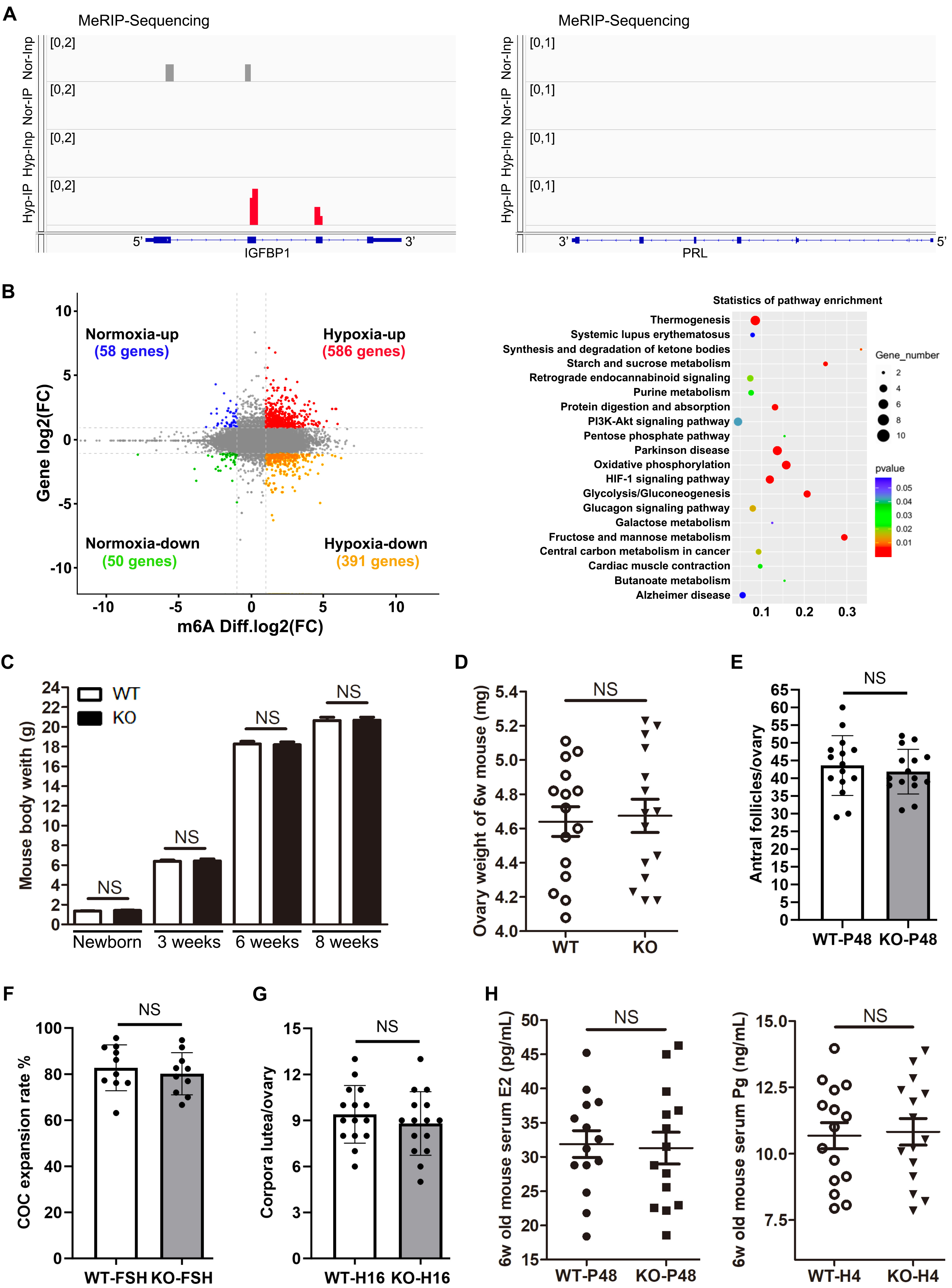

Supplement: Supplementary file 4 — Figure S3 [file CTM2-14-e1564-s004.jpg]

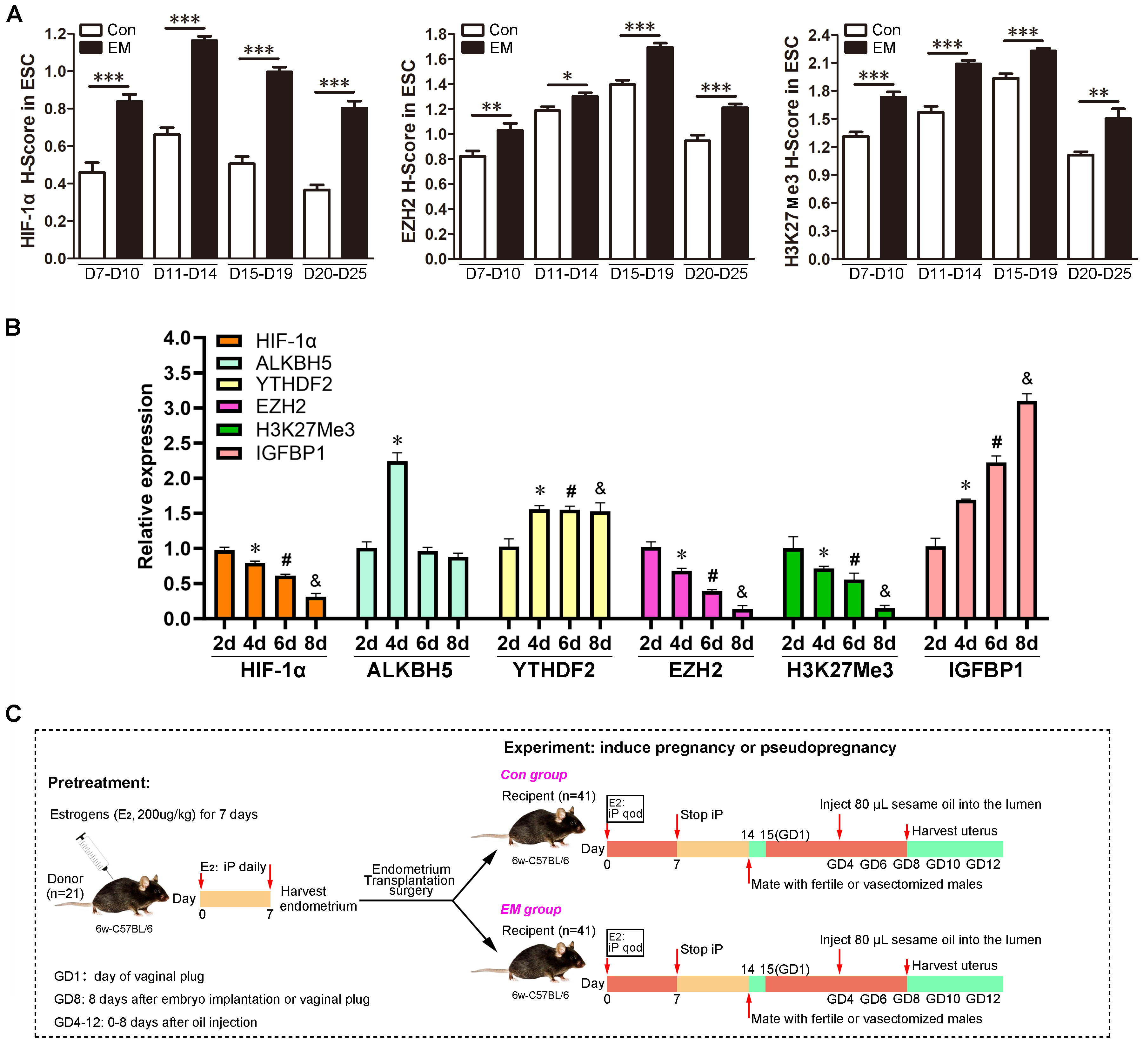

Supplement: Supplementary file 5 — Figure S4 [file CTM2-14-e1564-s002.jpg]

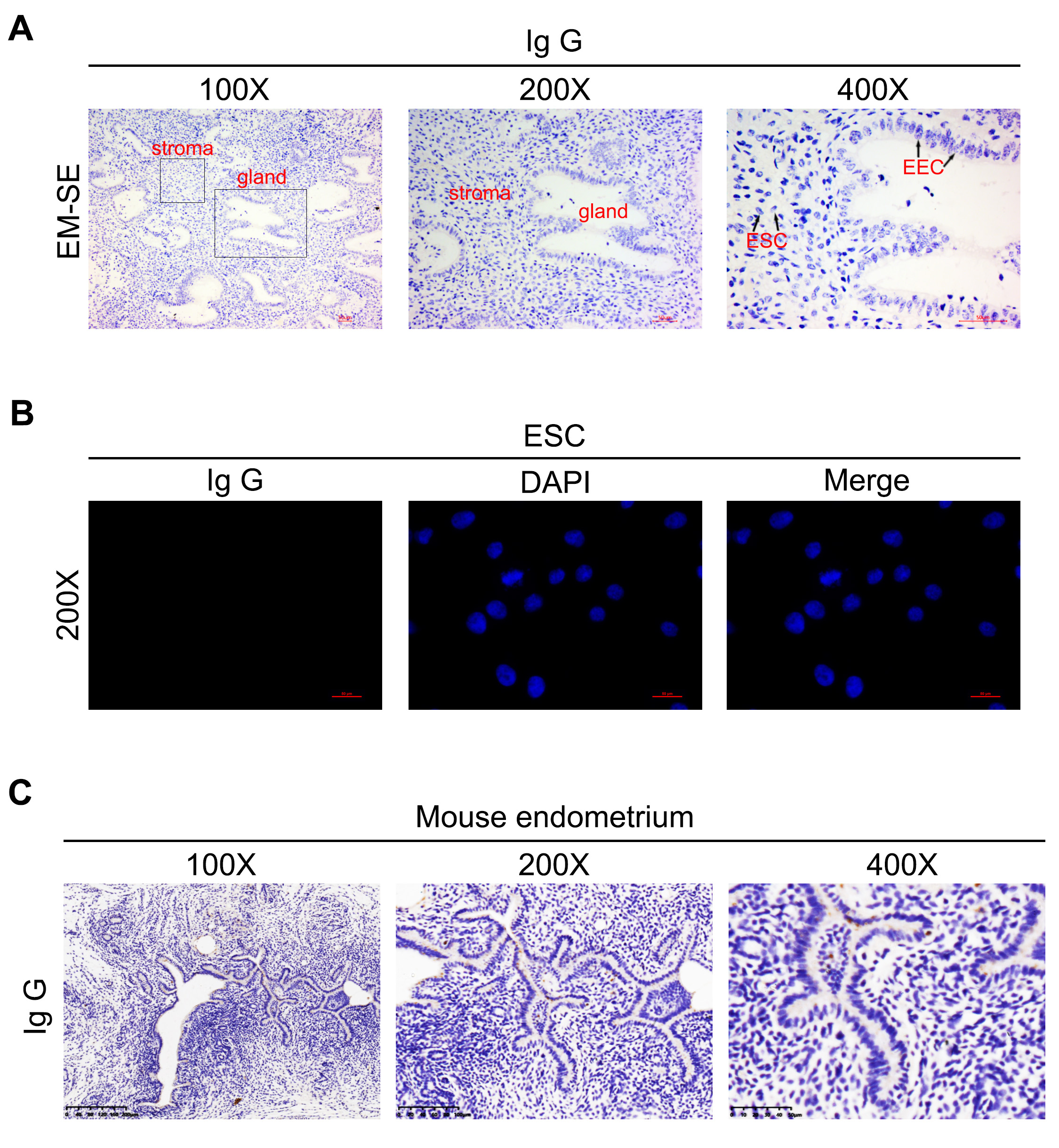

Supplement: Supplementary file 6 — Figure S5 [file CTM2-14-e1564-s001.jpg]
