# Supplementary material for: The involvement of RNA N6‐methyladenosine and histone methylation modification in decidualization and endometriosis‐associated infertility
Source: Clin Transl Med. 2024 Feb 12;14(2):e1564. doi: 10.1002/ctm2.1564 (PMC10859880; doi:10.1002/ctm2.1564)
Supplement: Supplementary file 7 — Supporting Information [file CTM2-14-e1564-s006.doc]

***Supplementary Information***

***Patient inclusion and exclusion criteria***

Endometriosis is defined as the presence of endometrium-like epithelium and/or stroma outside the uterine cavity, usually with an associated inflammatory process. Endometriosis in patients was confirmed by peritoneoscopy surgery followed by histological evidence. Endometriosis was classified as grades I–IV following the revised American Fertility Society classification system; we included both ovarian endometriosis and deep infiltrating endometriosis in our research.

Infertility was defined as failure to establish a clinical pregnancy after 1 year of regular, unprotected sexual intercourse or an impairment of a person’s capacity to reproduce either as an individual or with his/her partner. All patients in our study had undergone only one previous surgical excision of endometriotic lesions or salpingectomy and this was their first request for assisted reproductive therapy and embryo transfer. No patients received any hormones within 6 months.

The study inclusion criteria were as follows: (1) a history of infertility >1 year; (2) age 20–38 years; (3) basal serum follicle-stimulating hormone levels <8 IU/L and basal serum luteinizing hormone (LH) levels <10 IU/L; and (4) basic antral follicle count >6. Exclusion criteria included the following: (1) polycystic ovary syndrome or high androgen levels, premature ovarian failure, uterus diseases (e.g., uterine fibroids, Asherman’s syndrome, adenomyosis or endometrial polyp), diabetes, hypertension, thyroid diseases and other liver, kidney, heart or blood diseases; (2) any other untreated or insufficiently corrected endocrinopathies or immunopathy; and (3) smoking, alcoholism or drug addiction.

***Immunohistochemistry (IHC)***

IHC was performed as described before. After the corresponding treatment, tissues were fixed in 4% paraformaldehyde followed by embedding in paraffin for further hematoxylin and eosin (HE) staining or immunohistochemistry assay. Samples were serially sectioned at 5 mm thicknesses and then stained with HE. For IHC, slides were deparaffinized and rehydrated, and were incubated in the appropriate primary antibodies overnight at 4°C, followed by biotin-labeled secondary antibodies for 30 min. Staining procedure was performed using the Vectastain ABC kit and DAB peroxidase substrate kit (Vector Laboratories, Burlingame, CA, USA). Positive controls were included to confirm antibody specificity according to the instructions; and negative controls were performed by omitting the primary antibodies and using the respective IgGs.

A semi-quantitative grading system (H-score) was used to evaluate the intensity and percentage of IHC staining. This grading work was performed by two skilled technicians simultaneously. H-score of staining results was calculated using the following equation: H-score = Σ Pi (i + 1), where i = intensity of staining, with a value of 1, 2 or 3 (weak, moderate or strong, respectively), and Pi is the percentage of stained cells in total cells, ranging from 0 to 100%. The average H-score from two skilled technicians was used as the final staining score. Images were captured and analysed using an optic microscope (BX40, Olympus Optical Corporation, Tokyo, Japan).

***RNA isolation and real-time qPCR***

RNA isolation and real-time qPCR were performed as described before. All RNA concentrations were quantified using Nano-Drop 2000 software (Nanodrop, Wilmington, DE, USA). Complementary DNA (cDNA) was prepared from 1 ng RNA using a reverse transcription kit (R222-01, Vazyme, Nanjing, China). The abundance of mRNA was evaluated by qRT-PCR using SYBR (D7265, Beyotime Biotechnology, Shanghai, China). Quantitative RT-PCR was performed by 40 cycles of 95°C for 15 s and 60°C for 30 s.

RNA expression values were normalised to the arithmetic average of 18S rRNA and the quantification of mRNA abundance was based on the threshold cycle (Ct) as (Ct) as 2-Δ(ΔCt), whereΔCt=Ct( target gene)-Ct (18S) andΔ(ΔCt)= Ct(experimental group)-Ct(control group). The experiments were performed independently in triplicate in ESCs. The associated genes and their primers sequences are listed in **Table 1**.

***Western blot assay (WB)***

Western blot was performed as described before. Cells were freshly collected or scraped off plates, and total proteins were extracted from treated cells using radio-immunoprecipitation assay buffer (R0020, Solarbio, China) at 4°C. Protein concentration was determined using a bicinchoninic acid protein quantitative analysis kit (Pierce™ BCA Protein Assay Kit, 23225, Thermo Scientific™, MA, USA) according to the manufacturer’s instructions. Protein samples were separated by 10% SDS-PAGE and transferred to PVDF membranes (Bio-Rad, Hercules, CA, USA). The membranes were incubated in TBS supplemented with 5% skim milk powder at room temperature for 1 hour prior to incubation at 4°C overnight with the primary antibodies. The PVDF membranes were washed three times with TBS before incubation with HRP-labelled secondary antibodies (IRDye 800CW, LI-COR, USA) for 30-40 min at room temperature. Signals were developed with enhanced chemiluminescence (ECL) reagents (WBKLS0500, Millipore, Shanghai, China) and digitized on ChemiDoc™ Touch Imaging System (California, USA). Quantification of signals was carried out using Image J software. The experiments were performed independently in triplicate in ESCs. Positive controls were included to confirm antibody specificity according to the instructions; negative controls were performed by omitting the primary antibodies and using the respective IgGs. The primary antibodies and concentrations used in Western blot assay are listed in **Table 2**.

***Immunofluorescence assay (IF)***

To examine morphologic changes of human primary ESCs and explore the changes of EZH2 and H3K27Me3, immunofluorescence assay was performed using a standard staining procedure. In brief, primary cultured human ESCs were cultured on coverslips and fixed with 4% paraformaldehyde. After permeabilization with PBS-T (0.1% Triton X-100 in PBS solution), ESCs were blocked with 5% bovine serum albumin (BSA) for 30 min and then incubated with primary antibodies against phalloidine (1:1000, A12379, Thermo Scientific, USA), EZH2 (1:100, 5246, CST, USA), or H3K27Me3 (1:200, 9733, CST, USA) at 4°C overnight. Fluorescent-conjugated secondary antibody solution (1:100; MULTI SCIENCES, Hangzhou, China) was used to visualize the signal. Cell nuclei were visualized by staining with 4′,6-diamidino-2-phenylindole (DAPI, H-1200, Vector Laboratories, USA) solution. The primary antibodies and concentrations used in IF assay are listed in **Table 2**.

***Enzyme-linked immunosorbent assay***

Concentrations of human IGFBP-1 (EK1255-96, Multi Sciences Biotech, Hangzhou, China) and human prolactin (PRL, EK1304-96, Multi Sciences Biotech) in cell culture supernatants from the 13 human primary ESCs lines were measured using commercially available ELISA kits. Serum samples were collected through the epicanthus vein of mice. To examine the concentration of serum estrogen, paired WT or KO mice were treated by PMSG-48 h (n=14) first and then serum samples were collected through the epicanthus vein. To examine the concentration of serum progesterone, paired WT or KO mice were treated by PMSG-48 h followed by HCG-4 h (n=15).

All experiments were conducted according to the manufacturers’ instruction. The within and between assay coefficients of variation were 9.9% and 10.9%, respectively.

***Visualization of implantation sites and controlled ovarian hyperstimulation in mice.***

Six-week-old female mice were mated to fertile wild-type males and copulation was confirmed by the observation of vaginal plugs the following afternoon. The afternoon when the plug was observed was designated as GD1. Uteri were dissected at GD8, and implantation sites were photographed and counted.

For controlled ovarian hyperstimulation in mice, immature (3-week-old) or mature (6-week-old) female mice were injected intraperitoneally with 5 IU pregnant mare serum gonadotropin (PMSG, Ningbo San Sheng Biotech, Ningbo, China) for 48 h to stimulate follicle development, followed by injection with 5 IU human chorionic gonadotropin (HCG; Ningbo San Sheng Biotech) to induce ovulation. All mice were breed in accordance with the National Institutes of Health Guide for the Care and Use of Laboratory Animals. The Committee of Experimental Animal Ethics, Zhejiang University, approved the experiments.

***Ovulation analysis and hormone analysis***

Female mice were injected with 5 IU PMSG for 48 h followed by 5 IU HCG for 4–16 h. Ovaries were collected at PMSG 48 h (P48) for hematoxylin-eosin staining and antral follicles were counted. Cumulus-oocyte complexe (COC) were collected from the ovaries of immature WT or KO mice at P48. COC were plated in 50 µL defined COC medium under the cover of mineral oil and treated with 100 ng/mL FSH for 12 h. The expansion status was observed by microscopy. Corpora lutea were counted at hCG 16 h (H16).

Blood was collected by orbital venous puncture from female mice under isoflurane anesthesia. Serum was separated from the blood by centrifugation (3000 rpm for 10 minutes) and stored at −80 °C before hormone analysis. Serum E2 and Pg levels were measured at Sir Run Shaw Hospital, Zhejiang University School of Medicine.

***Flow cytometry assay for cell cycle distribution***

Human primary ESCs were starved for 12 h and then treated with normoxia (Con), hypoxia (Hyp), MPA+cAMP-based decidualization induction (Dec) or hypoxia and MPA+cAMP co-culture (DH) for 72 h. Cells were harvested gently, washed with cold PBS and fixed with ice-cold 70% ethanol overnight at 4°C. Cells were then washed twice with PBS and incubated with 0.5 ml PBS containing 50 µg/mL propidium iodide, 0.2% Triton X-100 and 100 µg/ml DNase-free RNase (Sigma-Aldrich, St. Louis, MO, USA) at room temperature in the dark for 30 minutes. The experiments were performed independently in triplicate (n=6). The results were analyzed using FACSCalibur (BD, CA, USA) and data were analyzed using Mod Fit LT 3.0 software.

***Flow cytometry assay for cell apoptosis***

Human primary ESCs were inoculated into 6 cm dishes at a density of 3x10^5^ cells/dish. At 12 h after adhesion, cells were cultured under normoxia, hypoxia, decidualization induction or hypoxia and MPA+cAMP co-culture for 72 h. An Annexin V‑FITC/propidium iodide (PI) apoptosis assay kit (cat. no. AD10; Dojindo Molecular Technologies, Inc.) was used to detect apoptotic cells. Trypsinized cells were washed twice with PBS and suspended in 100 µL 1X Annexin V binding solution. Next, 5 µL Annexin V‑FITC and PI staining solutions were added to the cell suspension, which were incubated at room temperature in the dark for 15 min. Following the addition of 400 µL 1X Annexin V binding solution, apoptosis was detected using ﬂow cytometry (CytoFLEX; Beckman Coulter, Inc.) and analyzed with CytExpert for DxFLEX (2.0.0.274; Beckman Coulter, Inc.). The apoptotic cell percentage (%) was calculated as Annexin V–positive cells/the total number of cells.

***Establishment of the endometriosis mouse model***

We established endometriosis mouse model to clarify the mechanism of RNA N6-methyladenosine and histone methylation modification *in vivo*. Six-week-old female C57BL/6 mice (23-25 g) were purchased from Shanghai Animal Centre, Chinese Academy of Science and housed in our animal center before initiating the experiments. Mice with regular 4 -5 days estrous cycles were used for further experiments. The allogeneic transplantation surgery for establishment of endometriosis mouse model is really common used and mature in our laboratory. Briefly, the 21 donor mice were pre-treated with 200 µg/kg 17β-estradiol (Sigma-Aldrich, St. Louis, MO, USA) daily by intraperitoneal injection for 7 days as presented in **Supplemental Figure. 3C**. The purpose of daily injection of estrogen to the donor mouse is to promote the growth of mouse endometrium. The donor uterus was excised from the uterine horn and cervix uteri, two strips were cut longitudinally with micro-scissors and the endometrial mucosa was exposed. Endometrial tissue with an approximate size of 0.5 cm × 0.3 cm was sutured on both ends using 6/0 polypropylene and then stitched to the bowel serosa of the intestinal mesentery. In parallel, 41 female C57BL/6 mice were sham-operated without implantation of endometrial tissue. To minimize the effect of the environment and surgical techniques, the operation time was limited to 15 min by Xiang Lin and Yongdong Dai. Two weeks after the transplantation, four mice were randomly selected from the Con or EM group to evaluate the endometriosis incidence of surgery. The implants grew into macroscopic ellipsoidal cysts that contained both endometrial glands and stroma in EM group.

Receptor mice were ovariectomized and 200 µg/kg 17β-estradiol every other day (qod) was administered for 7 days to support the implantation of ectopic cysts. In order to reduce the influence of estrogen on endometrium, a 1-week washout period was administered before mating with mates or artificially induced *in vivo* deciduoma as the effect of estrogen was believed to maintain less than14 days.

***References***

1. International working group of Aagl EE, Wes, Tomassetti C, Johnson NP, Petrozza J, Abrao MS, Einarsson JI, Horne AW, Lee TTM, Missmer S *et al*: **An International Terminology for Endometriosis, 2021**. *Journal of minimally invasive gynecology* 2021, **28**(11):1849-1859.

2. Zegers-Hochschild F, Adamson GD, Dyer S, Racowsky C, de Mouzon J, Sokol R, Rienzi L, Sunde A, Schmidt L, Cooke ID *et al*: **The International Glossary on Infertility and Fertility Care, 2017**. *Human reproduction* 2017, **32**(9):1786-1801.

3. Lin X, Dai Y, Tong X, Xu W, Huang Q, Jin X, Li C, Zhou F, Zhou H, Lin X *et al*: **Excessive oxidative stress in cumulus granulosa cells induced cell senescence contributes to endometriosis-associated infertility**. *Redox biology* 2020, **30**:101431.

4. Lin X, Dai Y, Xu W, Shi L, Jin X, Li C, Zhou F, Pan Y, Zhang Y, Lin X *et al*: **Hypoxia Promotes Ectopic Adhesion Ability of Endometrial Stromal Cells via TGF-beta1/Smad Signaling in Endometriosis**. *Endocrinology* 2018, **159**(4):1630-1641.

5. Dai Y, Lin X, Xu W, Lin X, Huang Q, Shi L, Pan Y, Zhang Y, Zhu Y, Li C *et al*: **MiR-210-3p protects endometriotic cells from oxidative stress-induced cell cycle arrest by targeting BARD1**. *Cell death & disease* 2019, **10**(2):144.

6. Fan HY, Liu Z, Johnson PF, Richards JS: **CCAAT/enhancer-binding proteins (C/EBP)-alpha and -beta are essential for ovulation, luteinization, and the expression of key target genes**. *Molecular endocrinology* 2011, **25**(2):253-268.

7. Fan HY, O'Connor A, Shitanaka M, Shimada M, Liu Z, Richards JS: **Beta-catenin (CTNNB1) promotes preovulatory follicular development but represses LH-mediated ovulation and luteinization**. *Molecular endocrinology* 2010, **24**(8):1529-1542.

8. Pelch KE, Sharpe-Timms KL, Nagel SC: **Mouse model of surgically-induced endometriosis by auto-transplantation of uterine tissue**. *Journal of visualized experiments : JoVE* 2012(59):e3396.

9. Zhou F, Zhao F, Huang Q, Lin X, Zhang S, Dai Y: **NLRP3 activated macrophages promote endometrial stromal cells migration in endometriosis**. *Journal of reproductive immunology* 2022, **152**:103649.

10. Galvankar M, Singh N, Modi D: **Estrogen is essential but not sufficient to induce endometriosis**. *Journal of biosciences* 2017, **42**(2):251-263.

11. Uegaki T, Taniguchi F, Nakamura K, Osaki M, Okada F, Yamamoto O, Harada T: **Inhibitor of apoptosis proteins (IAPs) may be effective therapeutic targets for treating endometriosis**. *Human reproduction* 2015, **30**(1):149-158.

12. Li J, Dai Y, Zhu H, Jiang Y, Zhang S: **Endometriotic mesenchymal stem cells significantly promote fibrogenesis in ovarian endometrioma through the Wnt/beta-catenin pathway by paracrine production of TGF-beta1 and Wnt1**. *Human reproduction* 2016, **31**(6):1224-1235.
